# Essential Domains of *Anaplasma phagocytophilum* Invasins Utilized to Infect Mammalian Host Cells

**DOI:** 10.1371/journal.ppat.1004669

**Published:** 2015-02-06

**Authors:** David Seidman, Kathryn S. Hebert, Hilary K. Truchan, Daniel P. Miller, Brittney K. Tegels, Richard T. Marconi, Jason A. Carlyon

**Affiliations:** Department of Microbiology and Immunology, Virginia Commonwealth University School of Medicine, Richmond, Virginia, United States of America; University of California, Los Angeles, UNITED STATES

## Abstract

*Anaplasma phagocytophilum* causes granulocytic anaplasmosis, an emerging disease of humans and domestic animals. The obligate intracellular bacterium uses its invasins OmpA, Asp14, and AipA to infect myeloid and non-phagocytic cells. Identifying the domains of these proteins that mediate binding and entry, and determining the molecular basis of their interactions with host cell receptors would significantly advance understanding of *A. phagocytophilum* infection. Here, we identified the OmpA binding domain as residues 59 to 74. Polyclonal antibody generated against a peptide spanning OmpA residues 59 to 74 inhibited *A. phagocytophilum* infection of host cells and binding to its receptor, sialyl Lewis x (sLe^x^-capped P-selectin glycoprotein ligand 1. Molecular docking analyses predicted that OmpA residues G61 and K64 interact with the two sLe^x^ sugars that are important for infection, α2,3-sialic acid and α1,3-fucose. Amino acid substitution analyses demonstrated that K64 was necessary, and G61 was contributory, for recombinant OmpA to bind to host cells and competitively inhibit *A. phagocytophilum* infection. Adherence of OmpA to RF/6A endothelial cells, which express little to no sLe^x^ but express the structurally similar glycan, 6-sulfo-sLe^x^, required α2,3-sialic acid and α1,3-fucose and was antagonized by 6-sulfo-sLe^x^ antibody. Binding and uptake of OmpA-coated latex beads by myeloid cells was sensitive to sialidase, fucosidase, and sLe^x^ antibody. The Asp14 binding domain was also defined, as antibody specific for residues 113 to 124 inhibited infection. Because OmpA, Asp14, and AipA each contribute to the infection process, it was rationalized that the most effective blocking approach would target all three. An antibody cocktail targeting the OmpA, Asp14, and AipA binding domains neutralized *A. phagocytophilum* binding and infection of host cells. This study dissects OmpA-receptor interactions and demonstrates the effectiveness of binding domain-specific antibodies for blocking *A. phagocytophilum* infection.

## Author Summary

*Anaplasma phagocytophilum* causes the potentially deadly bacterial disease granulocytic anaplasmosis. The pathogen replicates inside white blood cells and, like all other obligate intracellular organisms, must enter host cells to survive. Multiple *A*. *phagocytophilum* surface proteins called invasins cooperatively orchestrate the entry process. Identifying these proteins’ domains that are required for function, and determining the molecular basis of their interaction with host cell receptors would significantly advance understanding of *A*. *phagocytophilum* pathogenesis. In this study, the binding domains of two *A*. *phagocytophilum* surface proteins, OmpA and Asp14, were identified. The specific OmpA residues that interact with its host cell receptor were also defined. An antibody cocktail generated against the binding domains of OmpA, Asp14, and a third invasin, AipA, blocked the ability of *A*. *phagocytophilum* to infect host cells. The data presented within suggest that binding domains of OmpA, Asp14, and AipA could be exploited to develop a vaccine for granulocytic anaplasmosis.

## Introduction

Human granulocytic anaplasmosis (HGA) is an emerging tick-borne zoonosis in the United States, Europe, and Asia [1]. The number of HGA cases reported to the U. S. Centers for Disease Control and Prevention rose nearly seven-fold between 2003 and 2012 [2,3]. Seroprevalence data indicate that the disease is underreported in some endemic regions [4–8]. HGA can also be spread via perinatal, nosocomial, and blood transfusion routes [6,9–13]. It is an acute illness characterized by fever, chills, headache, malaise, leukopenia, thrombocytopenia, and elevated liver enzymes. Complications can include shock, seizures, pneumonitis, rhabdomyolysis, hemorrhage, increased susceptibility to secondary infections, and death. Risk for complications and fatality is greater for the elderly, the immunocompromised, and when proper diagnosis and/or antibiotic therapy are delayed [1]. The causative agent of HGA is *Anaplasma phagocytophilum*, an obligate intracellular bacterium that exhibits a tropism for neutrophils [1]. *A*. *phagocytophilum* is carried by a variety of wild animal reservoirs and, in addition to humans, causes disease in domestic animals including dogs, cats, horses, and sheep [14].

*A*. *phagocytophilum* exhibits a biphasic developmental cycle similar to that of *Chlamydia* spp., *Ehrlichia* spp., and *Coxiella burnetii* [15–18]. The *A*. *phagocytophilum* infectious dense-cored (DC) form promotes its receptor-mediated uptake into a host cell-derived vacuole. Within its vacuole, the DC develops into the non-infectious reticulate cell (RC) form that replicates to form a bacterial cluster called a morula [18,19]. RCs then convert back to DCs and are released to initiate the next infection cycle [18].

Sialyl Lewis x ([NeuAcμ(2–3)Galβ1–4(Fucα1–3)GlcNac]; sLe^x^), an α2,3-sialylated and α1,3-fucosylated core-2 O-linked glycan that caps the N-termini of selectin ligands [20], is a critical *A*. *phagocytophilum* receptor [21]. sLe^x^ is richly expressed on mammalian cells that are permissive for *A*. *phagocytophilum* infection—neutrophils, bone marrow progenitors, and promyelocytic HL-60 cells [22–24]. *A*. *phagocytophilum* recognizes sLe^x^ that caps the N-terminus of P-selectin glycoprotein ligand-1 (PSGL-1) on these myeloid cells [21,25]. Neutrophils and HL-60 cells that have been treated with an sLe^x^ blocking antibody, from which surface sialic acids have been enzymatically removed, or that are devoid of sialyltransferase and/or α1,3-fucosyltransferase activity are resistant to *A*. *phagocytophilum* binding and infection [19,21,26,27]. *A*. *phagocytophilum* also infects rhesus monkey choroidal (RF/6A) endothelial cells, megakaryoblastic MEG-01 cells, and bone marrow-derived mast cells in tissue culture. Infection of these non-myeloid host cell types depends on sLe^x^ itself, α2,3-sialic acid, and/or α1,3-fucose [28–35]. Thus, sLe^x^ and possibly other closely related α2,3-sialylated and α1,3-fucosylated molecules are essential for efficient *A*. *phagocytophilum* infection of mammalian cells.

We identified *A*. *phagocytophilum* OmpA and α2,3-sialic acid (N-acetylneuraminic acid [Neu5Ac], further referred to as sialic acid throughout) as the bacterium’s first adhesin/invasin-receptor pair [19]. OmpA binding to the α2,3-sialic acid determinant of sLe^x^ on myeloid cells and to α2,3-sialylated glycans on RF/6A cells are vital steps in *A*. *phagocytophilum* invasion of these host cell types [19]. Exposure of OmpA on the *A*. *phagocytophilum* DC surface makes it accessible to antibodies [19], which could be used to exploit the bacterium’s obligatory intracellular nature to block the host cell invasion step that is essential for survival. The OmpA binding domain that recognizes α2,3-sialic acid lies within amino acids 19 to 74 [19], but has yet to be specifically identified. The *A*. *phagocytophilum* OMP that recognizes α1,3-fucose is unknown. OmpA functions in concert with two additional invasins that are also upregulated during tick transmission feeding, Asp14 (14-kDa *A*. *phagocytophilum* surface protein) and AipA (*A*. *phagocytophilum* invasion protein A), to promote optimal *A*. *phagocytophilum* entry into mammalian host cells [29,36]. Thus, the most effective anti-granulocytic anaplasmosis approach may require targeting of all three invasins. We defined the AipA binding domain as residues 9 to 21 [36]. Pinpointing the OmpA and Asp14 binding domains; dissecting the interactions of key OmpA amino acids with α2,3-sialic acid and potentially α1,3-fucose; and evaluating the efficacy of targeting the OmpA, Asp14, and AipA binding domains together would potentially benefit development of approaches to block *A*. *phagocytophilum* infection.

In this study, we used antibody blocking, *in silico* docking models, and site-directed mutagenesis to identify the *A*. *phagocytophilum* OmpA binding domain, specifically the key residues that are essential for its adhesin/invasin activity, and determined that it recognizes both α2,3-sialic acid α1,3-fucose. This work represents the most detailed study of any rickettsial adhesin/invasin-receptor pair to date. Furthermore, we identified the Asp14 binding domain and confirmed that an antibody cocktail targeting the binding domains of OmpA, Asp14, and AipA nearly abolishes *A*. *phagocytophilum* infection of host cells.

## Results

### OmpA amino acids 59 to 74 are critical for *A*. *phagocytophilum* to bind to sLe^x^-capped PSGL-1 and for infection of mammalian host cells

**Fig 1:**
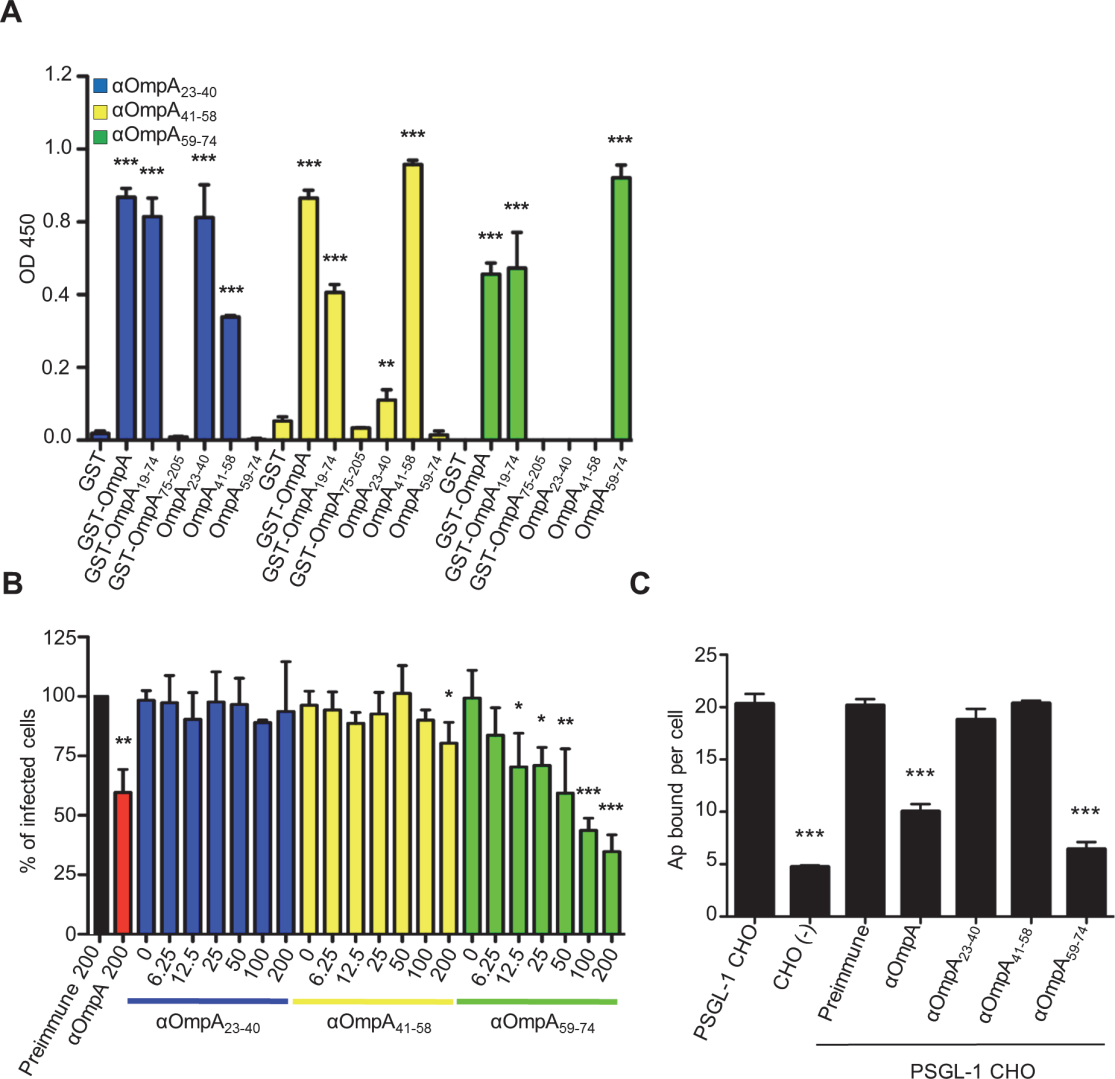
OmpA amino acids 59 to 74 are critical for *A*. *phagocytophilum* to bind to sLe^x^-capped PSGL-1 and for infection of mammalian host cells. (A) ELISA in which OmpA_23–40_, OmpA_41–58_, and OmpA_59–74_ antibodies (diluted 1:1600) were used to screen wells coated with GST, GST-OmpA, GST-OmpA_19–74,_ GST-OmpA_75–205_, or peptides corresponding to OmpA_23–40_, OmpA_41–58_, or OmpA_59–74_. Results shown are the mean ± SD of triplicate samples and are representative of three independent experiments with similar results. (B) Pretreatment of *A*. *phagocytophilum* with OmpA_59–74_ antibody inhibits infection of HL-60 cells in a dose-dependent manner. DC bacteria were incubated with 200 μg/ml of preimmune serum, 200 μg/ml of serum raised against GST-OmpA, or two-fold serially-diluted concentrations of sera raised against OmpA_23–40_, OmpA_41–58_, or OmpA_59–74_ ranging from 0 to 200 μg/ml and then incubated with HL-60 cells. The infection was allowed to proceed for 24 h after which the cells were fixed and examined using immunofluorescence microscopy to quantify the percentage of infected cells. Results shown are relative to host cells that had been incubated with bacteria exposed to preimmune serum and are representative of three experiments with similar results. (C) OmpA_59–74_ antibody inhibits *A*. *phagocytophilum* binding to sLe^x^-capped PSGL-1. DC bacteria were exposed to preimmune serum, antibodies against OmpA, OmpA_23–40_, OmpA_41–58_, or OmpA_59–74_ and then incubated with PSGL-1 CHO cells. Bacteria that were not exposed to antibodies and incubated with PSGL-1 CHO cells or CHO (-) cells were positive and negative controls, respectively, for bacterial binding. The mean numbers ± SD of bound DC organisms per cell were determined using immunofluorescence microscopy. Results shown are the mean ± SD of six combined experiments. Statistically significant (** *P* < 0.005; ***P < 0.001) values are indicated.

The OmpA region that is important for *A*. *phagocytophilum* infection of mammalian host cells lies within residues 19 to 74 (OmpA_19–74_) [19]. As a first step in further delineating the binding domain, we raised polyclonal antisera against peptides corresponding to OmpA amino acids 23 to 40, 41 to 58, and 59 to 74. We verified that the antisera were specific for OmpA by confirming that each recognized recombinant forms of mature OmpA (minus the signal sequence; corresponding to residues 19 to 205 and hereafter referred to as OmpA) and OmpA_19–74_, but neither OmpA_75–205_ nor Asp14 (S1 Fig. and Fig. 1A). Anti-OmpA_41–58_ and anti-OmpA_59–74_ were specific for their target peptides at all serum dilutions. Anti-OmpA_23–40_ was specific for its target peptide at most dilutions tested, but exhibited low level recognition of OmpA_41–58_ at dilutions below 1:12,800 (S1 Fig. and Fig. 1A). Next, we evaluated if any of the OmpA peptide antisera could inhibit *A*. *phagocytophilum* infection of host cells. Bacteria that had been treated with anti-OmpA or preimmune serum served as positive and negative controls, respectively. As previously observed [19], OmpA antibody reduced the percentage of *A*. *phagocytophilum* infected HL-60 cells by approximately 40% (Fig. 1B). OmpA_59–74_ antibody exhibited a dose-dependent inhibitory effect and, at a concentration of 200 ug/ml, reduced the percentage of infected HL-60 cells by approximately three-fold. Antisera targeting OmpA residues 23 to 40 and 41 to 58 exhibited very little to no inhibition of infection, regardless of concentration. Unless otherwise specified, all antisera were used at a concentration of 200 ug/ml in subsequent blocking experiments.

sLe^x^-capped PSGL-1 is an *A*. *phagocytophilum* receptor on human myeloid cells [21,25], and OmpA has been shown to bind the sLe^x^ portion [19]. Because OmpA_59–74_ antibody significantly inhibited *A*. *phagocytophilum* infection of HL-60 cells, we rationalized that OmpA amino acids that are critical for engaging the receptor are within residues 59 to 74. To test our hypothesis, we assessed the abilities of antisera targeting various portions of OmpA to interfere with *A*. *phagocytophilum* binding to Chinese hamster ovary cells transfected to express sLe^x^-capped PSGL-1 (PSGL-1 CHO cells) [37]. These cells are useful models for studying *A*. *phagocytophilum* interactions with sLe^x^ and/or PSGL-1 because they robustly support bacterial binding but not infection, while untransfected CHO [CHO (-)] cells that lack expression of these receptors poorly support bacterial binding [18,19,26,27,29]. Anti-OmpA_59–74_ reduced the mean number of bound *A*. *phagocytophilum* DC organisms per PSGL-1 CHO cell by approximately four-fold to nearly that of CHO (-) cells (Fig. 1C). Anti-OmpA reduced bacterial binding to PSGL-1 CHO cells by approximately two-fold. Anti-OmpA_23–40_, anti-OmpA_41–58_, and preimmune serum had no effect. These results indicate that the OmpA binding domain lies within amino acids 59 to 74 and this region is important for *A*. *phagocytophilum* recognition of sLe^x^-capped PSGL-1.

### Molecular docking models of *A*. *phagocytophilum* OmpA-sLe^x^ interactions suggest that residues within OmpA_59–74_ engage sLe^x^

**Fig 2:**
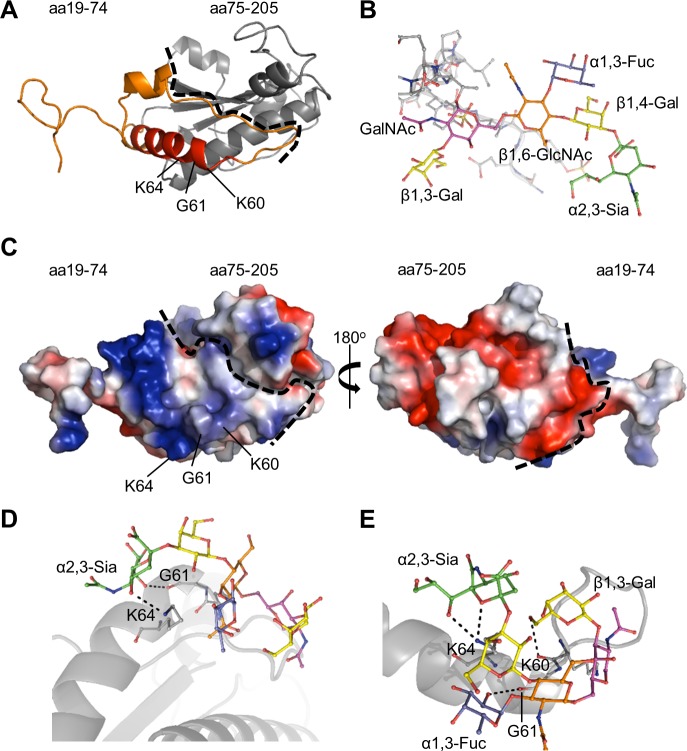
Molecular docking models of *A*. *phagocytophilum* OmpA-sLe^x^ interactions. (A) Predicted tertiary structure for *A*. *phagocytophilum* OmpA. The orange portion delineates residues 19 to 74. The red portion corresponds to amino acids 59 to 74, and the gray portion corresponds to residues 75 to 205. The dotted line separates the regions encompassed by residues 19 to 74 and 75 to 205. Residues K60, G61, and K64 positions are indicated. (B) Stick representation of the N-terminal PSGL-1 amino acids 61 to 77 (gray) capped with sLe^x^ derived from PDB 1g1s. The sLe^x^ glycan extends off of threonine 73. sLe^x^ linkages and individual sugar residues are denoted. (C) Electrostatic surface map of *A*. *phagocytophilum* OmpA, as generated using the PyMol APBS plugin. The left image is oriented as in (A). The right image is rotated 180° around the y-axis. Positive and negative charges are indicated by blue and red, respectively. The dotted line is a demarcation between the regions encompassed by residues 19 to 74 and 75 to 205, which have overall cationic and anionic surface charges, respectively. (D and E) OmpA and sLe^x^ interactions predicted by the Autodock Vina algorithm. OmpA is presented as a gray ribbon model, sLe^x^ as a multicolor stick model, and hydrogen bonding by dotted lines. OmpA residue K64 is predicted to interact with α2,3-sialic acid (green) of sLe^x^ (D and E). Residue G61 is predicted to interact with either α2,3-sialic acid (D) or α1,3-fucose (blue) of sLe^x^ (E). Residue K60 is predicted to interact with β1,3-galactose of sLe^x^ (E).

To complement our antibody blocking experiments, molecular modeling and docking was used to identify the OmpA amino acids that possibly contact sLe^x^. First, a three-dimensional model of the invasin was generated. A crystal structure for *A*. *phagocytophilum* OmpA has yet to be determined, but an abundance of crystal structures for similar bacterial proteins have. The Phyre2 (Protein Homology/ Analogy Recognition Server version 2.0) server (www.sbg.bio.ic.ac.uk/phyre2), which predicts three-dimensional structures for protein sequences and threads the predicted models on known crystal structures [38], was used to generate a tertiary structure model for OmpA (Fig. 2A). The resulting homology model predicted that OmpA residues 59 to 74 form part of a surface-exposed alpha helix (Fig. 2A), which could potentially interact with ligands. Surface electrostatic values calculated using the adaptive Poisson-Boltzmann solver (APBS) [39] plugin for PyMOL (pymol.org/educational) indicated that OmpA amino acids 19 to 74 have an overall cationic surface charge. The rest of the modeled protein exhibits an overall anionic surface charge (Fig. 2C). These findings are consistent with prior observations that bacterial and viral proteins that interact with sLe^x^ and/or sialic acid do so at cationic surface patches [40–44].

For docking predictions, the sLe^x^ glycan (Fig. 2B) was extracted from the crystal structure of sLe^x^-capped PSGL-1 (DOI:10.2210/pdb1g1s/PDB). Autodock Vina was used to predict how OmpA might interact with sLe^x^ [45,46]. The search grid encapsulated OmpA_19–74_ (Fig. 2A). The top two docking models, each with the same predicted affinity value of -4.2 kcal/mol, displayed similar interactions between sLe^x^ and the OmpA region encompassed by amino acids 59 to 74. In both models, K64 of OmpA was predicted to bind the α2,3-sialic acid residue of sLe^x^ (Fig. 2, D and E). G61 was also predicted to interact with sLe^x^ in both models, though it was predicted to bind α2,3-sialic acid in one model and α1,3-fucose in the other. Lastly, K60 was predicted to bind the ß1,3-galactose residue of sLe^x^ in the docking model presented in Fig. 2E. Together, the *in silico* predictions and peptide antibody blocking results suggest that OmpA_59–74_ contains critical residues that interact with sLe^x^ to promote *A*. *phagocytophilum* infection of host cells.

### OmpA is conserved among *A*. *phagocytophilum* strains and K64 is conserved among *Anaplasmataceae* OmpA proteins

Aligning the OmpA sequence from the *A*. *phagocytophilum* NCH-1 strain that we study, which was originally isolated from a HGA patient in Nantucket, MA [47], with those encoded by geographically diverse *A*. *phagocytophilum* isolates that had been recovered from infected humans, animals, and ticks [48–54] revealed that OmpA is highly conserved among these strains (S2A Fig.). Eight of the nine sequences were identical. The OmpA of NorV2 Norwegian sheep isolate [53] had only three amino acid differences, none of which were within the binding domain encompassed by residues 59 to 74. The high degree of OmpA sequence conservation further supports the invasin’s importance to *A*. *phagocytophilum* pathobiology. We next aligned NCH-1 OmpA residues 19 to 74 with corresponding regions of OmpA homologs from *A*. *marginale* and *Ehrlichia* spp., which are in the family *Anaplasmataceae* with *A*. *phagocytophilum* and infect bovine erythrocytes and human and animal monocytes, respectively [55–57]. *A*. *phagocytophilum* OmpA K64 that was predicted to potentially interact with sLe^x^ (Fig. 2, D and E), was the only binding domain residue that was conserved among all *Anaplasmataceae* OmpA regions examined (S2B Fig.). Additional residues within the *A*. *phagocytophilum* OmpA binding domain, including the other two predicted to interact with sLe^x^, K60 and G61 (Fig. 2, D and E), were conserved among *Anaplasma* spp. but not *Ehrlichia* spp. OmpA proteins (S2B Fig.).

### G61 and K64 are essential for recombinant OmpA to optimally bind to mammalian host cells and competitively inhibit *A*. *phagocytophilum* infection

**Fig 3:**
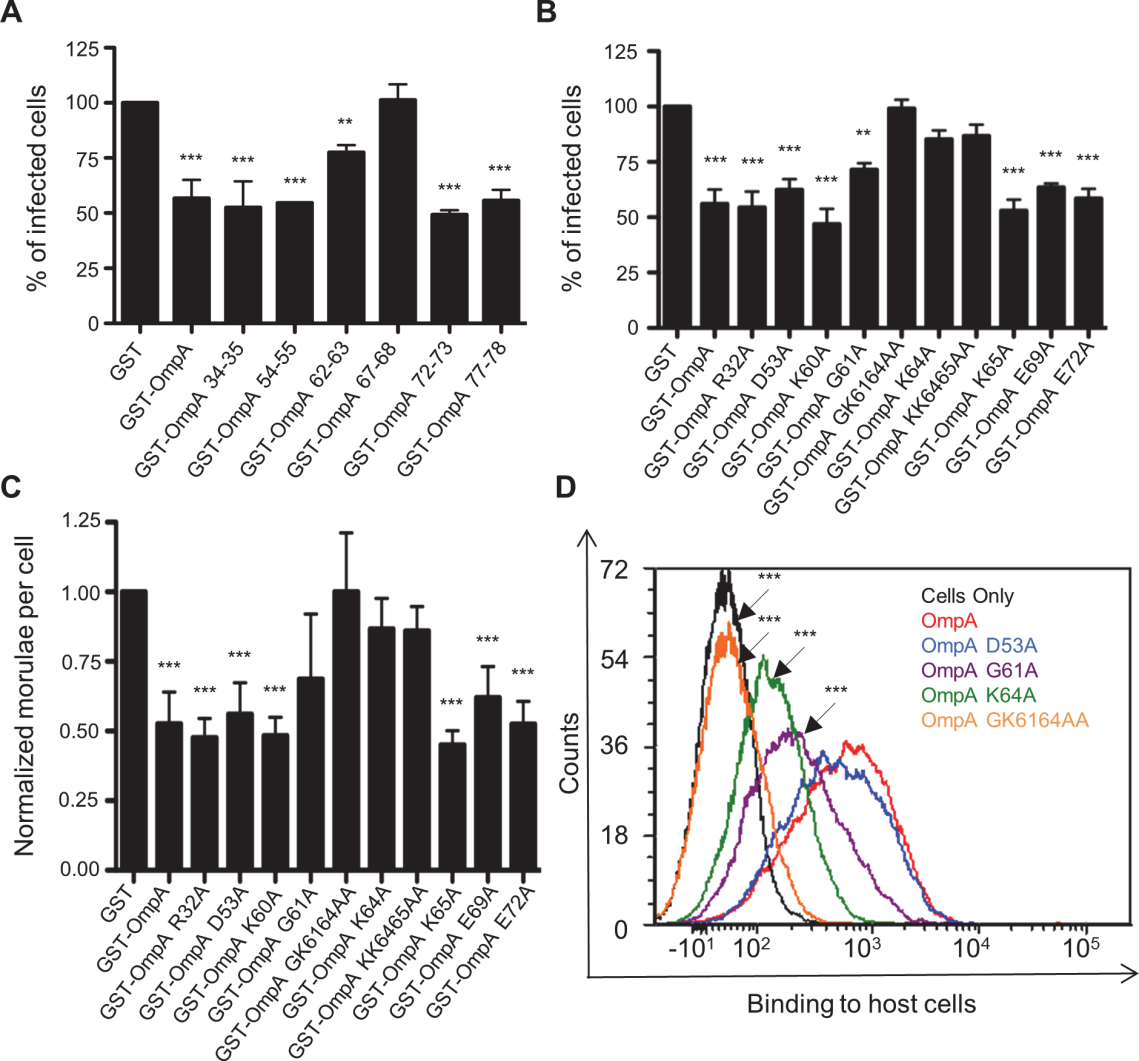
G61 and K64 are essential for recombinant OmpA to optimally bind to mammalian host cells and competitively inhibit *A*. *phagocytophilum* infection. GST-OmpA proteins having the CLNHL peptide inserted between OmpA amino acids 67 and 68 or having G61 and/or K64 mutated to alanine are unable to bind to competitively inhibit *A*. *phagocytophilum* infection of mammalian host cells. HL-60 cells were incubated with DC organisms in the presence of GST alone, GST-OmpA, GST-OmpA proteins bearing insertions of CLNHL between the indicated residues (A), or GST-OmpA proteins having the indicated amino acids substituted with alanine (B and C) for 1 h. After washing to remove unbound bacteria, host cells were incubated for 24 h and subsequently examined by immunofluorescence microscopy to determine the percentage of infected cells (A and B) or the mean number (± SD) of morulae per cell (C). Results shown in (A), (B), and (C) are the means ± SD for six to twelve combined experiments. The data presented in panel C are the normalized values of six to twelve experiments. Statistically significant (** *P* < 0.005; ***P < 0.001) values are indicated. (D) Flow cytometric analysis of His-OmpA and His-OmpA proteins bearing alanine substitutions binding to RF/6A cells. Data are representative of two experiments with similar results.

Because *A*. *phagocytophilum* is an obligate intracellular bacterium, developing a knock out-complementation system for this organism has proved challenging and has not been described. Therefore, we utilized a series of alternative approaches to further functionally evaluate OmpA. Recombinant OmpA can be used as a competitive agonist to block *A*. *phagocytophilum* access to its receptor and thereby inhibit infection [19]. We exploited this phenomenon to further define the OmpA amino acids that are critical for receptor recognition and bacterial uptake by assessing the competitive agonist abilities of OmpA proteins having site-directed amino acid changes. Our approach was built on the rationale that OmpA proteins in which the binding domain was disrupted would be unable to inhibit infection. First, we generated OmpA proteins N-terminally fused to glutathione-*S*-transferase (GST), each of which had an insertion of the peptide CLNHL at one of six different sites within residues 19 to 78. This approach has been used in previous studies to disrupt proteins’ binding domains without perturbing overall protein structure, and the insertion sequence that we devised for this purpose was a consensus of the insertion peptides used in those studies [58–60]. Incubating HL-60 cells with the positive control, GST-OmpA, prior to the addition of DC bacteria resulted in a significant reduction in the percentage of infected cells relative to GST alone (Fig. 3A), as shown previously [19]. GST-OmpA proteins carrying insertions between residues 67 and 68 and between 62 and 63 were completely and partially abrogated, respectively, in their abilities to inhibit *A*. *phagocytophilum* infection. GST-OmpA proteins bearing insertions at other sites were unaffected in their ability to inhibit infection.

We next set out to identify the specific amino acids of GST-OmpA that were critical for it to inhibit *A*. *phagocytophilum* infection. We repeated the competitive agonist assay using GST-OmpA proteins in which select amino acids had been mutated to alanine (S2 Fig. and Fig. 3, B and C). Many of the targeted residues were within OmpA amino acids 59 to 74. R32 and D53 were selected because they lie outside of residues 59 to 74, and, accordingly, we anticipated that substituting them would not alter OmpA function. GST-OmpA_K64A_ was considerably reduced in its ability to inhibit *A*. *phagocytophilum* infection (Fig. 3, B and C), thereby indicating that this highly conserved residue was critical for GST-OmpA to serve as a competitive agonist. K65, however, was dispensable for this function, as the blocking ability of GST-OmpA_K65A_ was uncompromised and the blocking ability of GST-OmpA_KK6465AA_ was no greater than that of GST-OmpA_K64_. GST-OmpA_G61A_ displayed a modest but significant decline in its competitive agonist ability. Replacement of both G61 and K64 with alanines yielded an additive effect that was greater than substituting either residue alone, as GST-OmpA_GK6164AA_ was unable to inhibit infection. GST-OmpA proteins in which R32, D53, K60, E69 and E72 had been mutated to alanine were each unaffected in the ability to hinder infection.

Given that K64 and G61 are vital and contributory, respectively, to the ability of recombinant OmpA to competitively inhibit *A*. *phagocytophilum* infection, we evaluated if these residues mediate binding to mammalian host cell surfaces. RF/6A and HL-60 cells were incubated with His-tagged OmpA proteins. After unbound proteins were washed away, bound proteins were detected by flow cytometry using a His-tag antibody. His-tagged OmpA and OmpA_D53A_ bound equally well to RF/6A cells (Fig. 3D). His-OmpA_G61A_ bound poorly, His-OmpA_K64A_ even more so, and His-OmpA_GK6164AA_ could not bind to host cells. Collectively, these data are consistent with the invasin-receptor contacts predicted by the OmpA-sLe^x^ docking models and underscore the importance of OmpA K64 and G61 to OmpA-receptor interactions.

### OmpA interacts with α1,3-fucose on mammalian host cell surfaces

α1,3-fucose is critical for *A*. *phagocytophilum* to bind PSGL-1-modeled glycopeptides, to bind and invade human and murine myeloid cells, and to establish infection in laboratory mice [25–27]. Consistent with these observations, PSGL-1 CHO cells that had been pretreated with α1,3/4-fucosidase were approximately three-fold less permissive for *A*. *phagocytophilum* binding (S3A Fig.). Multiple lines of evidence led us to hypothesize that OmpA binds α1,3-fucose. First, OmpA binds α2,3-sialic acid [19], which is in close proximity to α1,3-fucose on sLe^x^ [46]. Second, the docking model in Fig. 2E predicted that OmpA residues within the binding domain contact both α2,3-sialic acid and α1,3-fucose of sLe^x^. Third, OmpA is important for *A*. *phagocytophilum* infection of not only myeloid, but also endothelial cells [19]. Fourth, fucose residues are critical for the pathogen to invade RF/6A endothelial cells, as pretreatment of the host cells with α1,3/4-fucosidase made them significantly less permissive to *A*. *phagocytophilum* binding (S3B Fig.) and infection (S3C Fig.).

**Fig 4:**
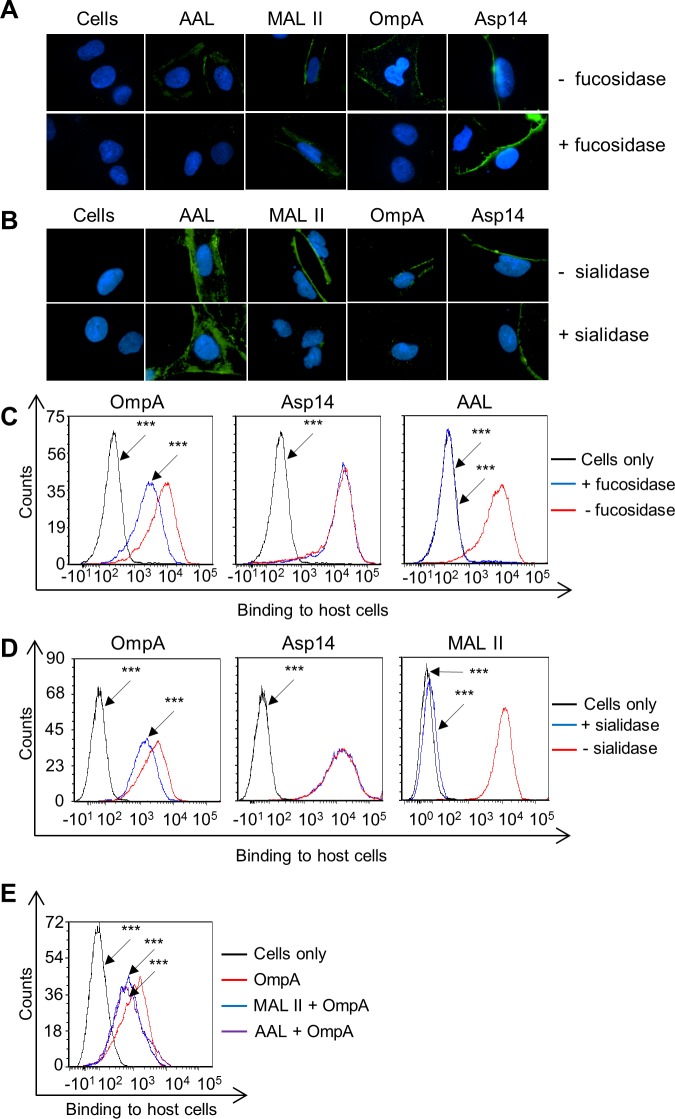
OmpA interacts with α1,3-fucose on mammalian host cell surfaces. (A to D) RF/6A cells were treated with α1,3/4-fucosidase (A and C), α2,3/6-sialidase (B and D), or vehicle control (- fucosidase and—sialidase, respectively). Glycosidase- and mock-treated cells were incubated with α1,3/6-fucose-specific lectin, AAL; the α2,3-sialic acid-specific lectin, MAL II; His-OmpA; or His-Asp14. (A) The host cells were fixed and screened using immmunofluorescence microscopy (A and B) or flow cytometry (C and D) to detect lectin, His-OmpA, or His-Asp14 binding to host cells. In A and B, green fluorescence corresponds to lectin (AAL or MAL II) or His-tagged protein (OmpA or Aps14) bound at cell surfaces. Host cell nuclei are stained blue by DAPI. (E) AAL and MAL II competitively inhibit His-OmpA binding to mammalian host cells. RF/6A cells were incubated with AAL and MAL II, after which His-OmpA was added. Following the removal of unbound recombinant protein, His-OmpA bound on RF/6A cell surfaces was detected by flow cytometry. Statistically significant (***P < 0.001) values are indicated. Results shown are representative of three experiments with similar results.

To determine if OmpA recognizes fucose, His-tagged OmpA was incubated with RF/6A cells that had been treated with α1,3/4-fucosidase and binding was assessed by immunofluorescence microscopy and flow cytometry. α2,3/6-sialidase-treated RF/6A cells were included as a positive control for a treatment that would make the host cells less permissive to recombinant OmpA binding [19]. To verify the efficacy and specificity of both glycosidases, treated and untreated host cells were screened with AAL (*Aleuria aurantia* lectin) and MAL II (*Maackia amurensis* lectin II). AAL recognizes fucose residues that are in α1,3- and α1,6-linkages with N-acetylglucosamine [61,62]. MAL II detects sialic acids that are in α2,3-linkages with galactose [63]. Fucosidase treatment abolished AAL but not MAL II binding, while sialidase treatment eliminated MAL II but not AAL binding (Fig. 4, A to D). Thus, the glycosidases were effective and specific. His-OmpA binding to both sialidase- and fucosidase-treated RF/6A cells was comparably reduced relative to vehicle control treated cells, while binding of His-Asp14 was unaffected. Incubating the host cells with MAL II or AAL prior to the addition of His-OmpA competitively reduced the efficiency of His-OmpA binding by similar degrees as sialidase or fucosidase, respectively (Fig. 4E). Overall, these observations demonstrate that optimal adhesion of OmpA to host cells involves both α2,3-sialic acid and α1,3-fucose and that Asp14 utilizes neither sialic acid nor fucose to bind to host cells.

### OmpA interacts with 6-sulfo sLe^x^ on RF/6A endothelial cell surfaces

**Fig 5:**
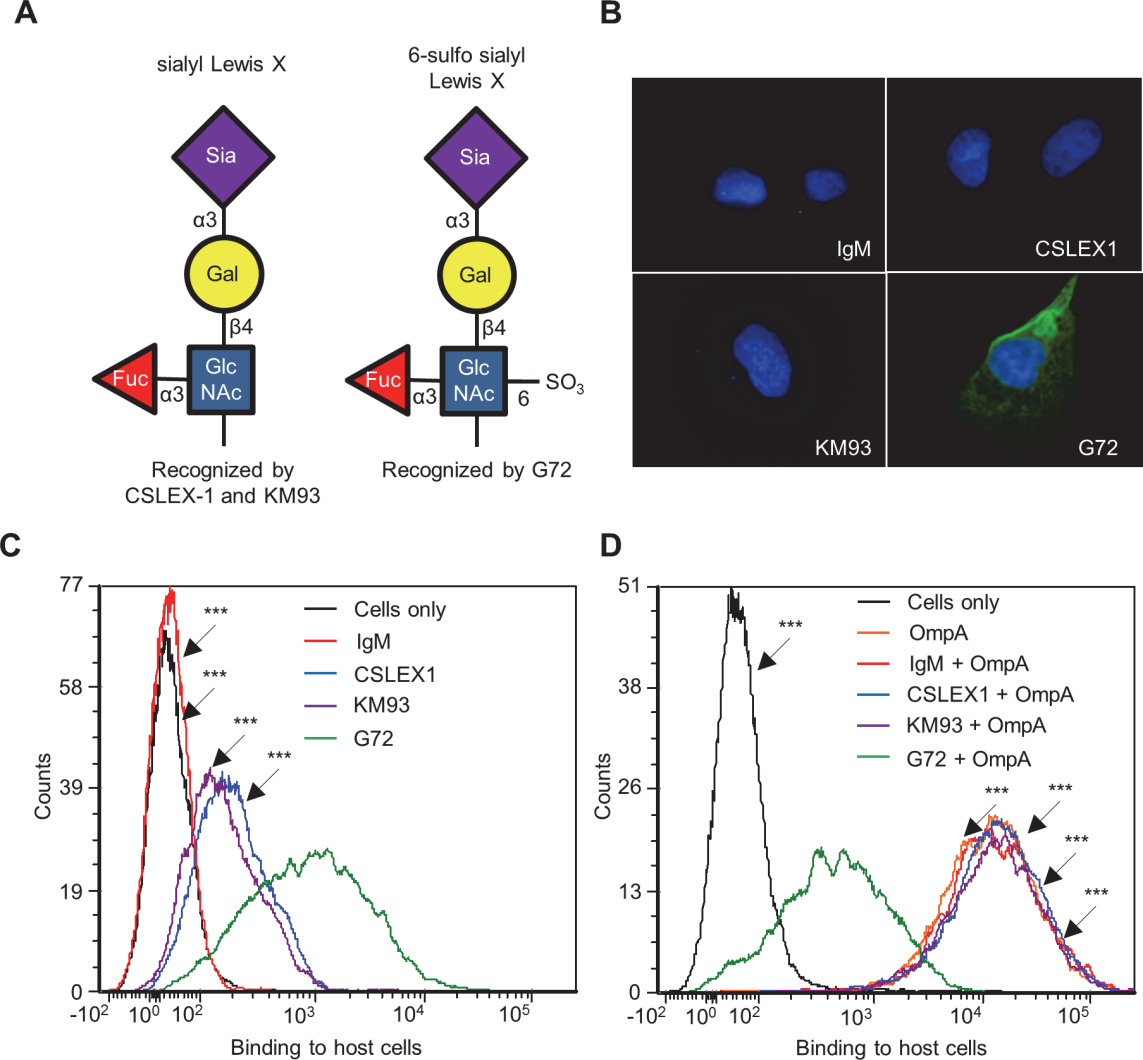
OmpA interacts with 6-sulfo sLe^x^ on RF/6A endothelial cell surfaces. (A) Schematic representations of sLe^x^ and 6-sulfo sLe^x^. Below each diagram is a statement denoting monoclonal antibodies that recognize each tetrasaccharide. Individual sugar and glycosidic linkages are indicated. (B and C) 6-sulfo sLe^x^ is present in high abundance relative to sLe^x^ on RF/6A cells. RF/6A cells were screened with sLe^x^-specific antibodies, CSLEX1 and KM93; 6-sulfo sLe^x^-specific antibody, G72; or IgM isotype control followed by detection of cell surface bound antibodies using immunofluorescence microscopy (B) and flow cytometry (C). (D) Antibody blocking of 6-sulfo sLe^x^ inhibits His-OmpA binding to RF/6A cells. RF/6A cells were incubated with CSLEX1, KM93, G72, IgM, or vehicle (Cells only) followed by the addition of His-OmpA, and washing to remove unbound recombinant protein. Flow cytometry was used to detect bound His-OmpA. Statistically significant (***P < 0.001) values are indicated. Results shown are representative of two experiments with similar results.

Because His-OmpA binding to RF/6A cells involved recognition of α2,3-sialic acid and α1,3-fucose (Fig. 4), we hypothesized that OmpA interacts with sLe^x^ or a sLe^x^-like receptor on these host cells. sLe^x^ and the sLe^x^-like molecule, 6-sulfo sLe^x^ (Neu5Ac(α2–3)Gal(β1–4)[Fuc(α1–3)][HSO_3_(3–6)]GlcNAc1) (Fig. 5A) have both been detected on the surfaces of high endothelial venal, vascular, cancerous, and/or inflamed endothelial cells [64–76]. To assess if either glycan is present on RF/6A cells, we screened them with sLe^x^ antibodies, CSLEX1 [77] and KM93 [78] and the 6-sulfo-sLe^x^ antibody, G72 [64]. Robust G72 signal but little to no CSLEX1 or KM93 signal was detected on RF/6A cells (Fig. 5, B and C). Binding of His-OmpA to RF/6A cells that had been pretreated with G72 was pronouncedly reduced relative to cells that had been incubated with CSLEX1, KM93, or isotype control antibody (Fig. 5D). Thus, *A*. *phagocytophilum* OmpA recognizes 6-sulfo-sLe^x^ on RF/6A endothelial cells.

### OmpA-coated beads bind to and are internalized by non-phagocytic endothelial cells

**Fig 6:**
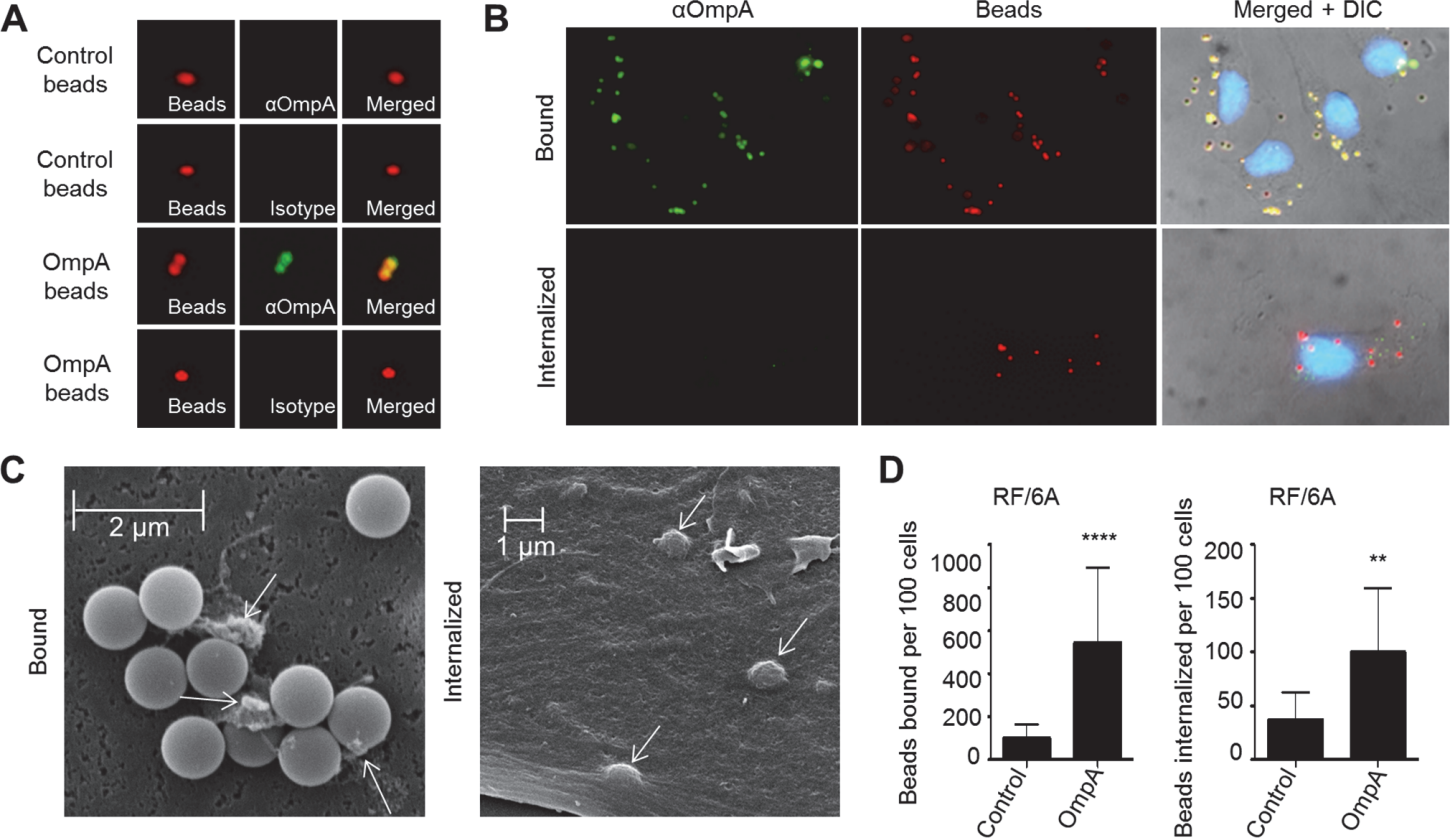
OmpA coated beads bind to and are internalized by non-phagocytic endothelial cells. (A) Confirmation of recombinant OmpA conjugation to inert beads. Red fluorescent OmpA-coated microspheres (OmpA beads) were incubated with OmpA antibody (αOmpA) or isotype control. Unconjugated (Control) beads were included as a negative control. Bound antibody (green) was detected using immunofluorescence microscopy. OmpA coated beads with bound antibody appeared yellow when the individual fluorescence channels were merged, whereas beads without bound antibody appeared red. (B to D) OmpA coated beads bind to and are internalized by non-phagocytic RF/6A cells. OmpA coated beads were incubated with RF/6A cells for 1 h after which unbound beads were washed off. Cells were screened with OmpA antibody and examined using immunofluorescence (B and D) or scanning electron microscopy (C) to assess binding or were incubated further to allow for bead uptake. To assess for internalized beads, the host cells were treated with trypsin, washed, incubated overnight for the endothelial cells to re-adhere, fixed, and screened with OmpA antibody and immunofluorescence microscopy. Because the host cells were not permeabilized, bound beads detected by OmpA antibody appeared yellow and internalized beads, which OmpA antibody could not detect, remained red when viewed by immunofluorescence microscopy. (B) DIC, differential interference contrast microscopy. (C) Two scanning electron micrographs depicting bound or internalized OmpA coated beads. Arrows denote filopodia-like structures bound to beads. Scale bars are indicated. Results in (D) are representative of thirteen experiments with similar results. Statistically significant (** *P* < 0.005; ****P < 0.0001) values are indicated.

The ability of recombinant OmpA to bind to non-phagocytic RF/6A endothelial cells [19] (Figs. 3–5), suggests that, in addition to functioning as an invasin, it may also exhibit adhesin activity. Furthermore, while OmpA on the *A*. *phagocytophilum* surface acts cooperatively with Asp14 and AipA to mediate bacterial binding to and invasion of mammalian host cells [19,29,36], its ability to mediate these processes by itself is unknown. Therefore, we assessed the ability of recombinant OmpA to confer adhesiveness and invasiveness to inert particles. His-OmpA was coupled to red fluorescent microspheres that were 1.0 μm in diameter, a size similar to that of the diameter of an *A*. *phagocytophilum* DC organism (0.8 ± 0.2 μm) [18]. Successful conjugation of His-OmpA to the beads was confirmed by immunofluorescence using OmpA antiserum (Fig. 6A). RF/6A cells were incubated with recombinant OmpA-coated or non-coated control beads and screened with OmpA antibody to determine the numbers of beads bound per cell. To assess bead uptake, the cells were incubated for an additional 1 to 8 h and trypsin was used to remove non-internalized beads prior to screening. Immunofluorescence microscopy revealed that significantly more OmpA coated beads bound to and were internalized by RF/6A cells versus control beads (Fig. 6, B and D). Scanning electron microscopy corroborated these results, as OmpA coated bead were observed bound to and inducing the formation of filopodia-like structures on the surfaces of RF/6A cells or covered by plasma membrane (Fig. 6C). Thus, OmpA alone was sufficient to mediate bead binding to and uptake by non-phagocytic RF/6A endothelial cells.

### Binding and uptake of OmpA-coated beads by myeloid cells is dependent on sLe^x^

**Fig 7:**
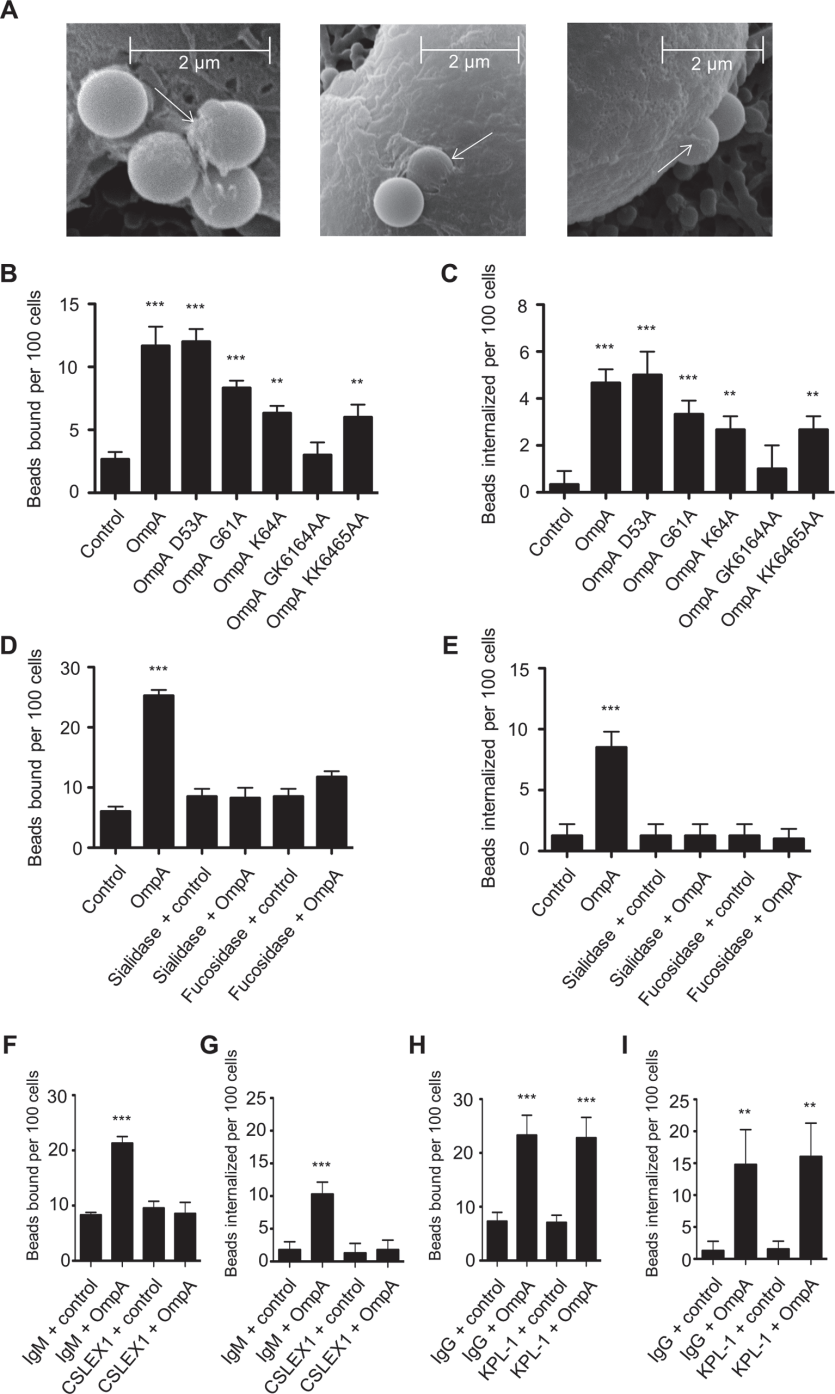
OmpA coated bead binding to and uptake by promyelocytic HL-60 cells involve OmpA residues G61 and K64 and are dependent on sLe^x^. (A) Scanning electron micrographs depicting OmpA coated beads bound to and being internalized by HL-60 cells. Arrows point to filopodia-like structures adhered to beads. Scale bars are indicated. (B and C) HL-60 cells were incubated with beads coated with OmpA, OmpA proteins having the indicated amino acids substituted with alanine, or non-coated control beads. The numbers of bound and internalized beads were determined using immunofluorescence microscopy. (D to I) HL-60 cells were incubated with α2,3/6-sialidase, α1,3/4-fucosidase, or vehicle only (D and E), sLe^x^ antibody CSLEX1 or IgM isotype control (F and G), or PSGL-1 N-terminus antibody KPL-1 or IgG isotype control (H and I) before being incubated with OmpA coated or non-coated control beads. The numbers of bound and internalized beads were assessed using immunofluorescence microscopy. Results in (B) through (I) are the mean ± SD of triplicate samples and are representative of three independent experiments with similar results. Statistically significant (** *P* < 0.005; ***P < 0.001) values are indicated.

We next assessed the ability of His-OmpA coated beads to bind and enter HL-60 cells and, if so, whether these processes involve the OmpA myeloid cell receptor, sLe^x^. Scanning electron microscopy revealed that OmpA beads bound to and induced their own uptake into HL-60 cells (Fig. 7A). Relative to the results obtained using RF/6A cells (Fig. 6D), OmpA coated bead binding to HL-60 cells was reduced (Fig. 7B). However, of the OmpA beads that did bind, approximately half of them were internalized (Fig. 7C). Approximately three-fold fewer control beads than OmpA coated beads bound to and were taken in (Fig. 7, B and C). OmpA bead uptake, but not adherence was pronouncedly inhibited when the assay was performed at 4°C versus 37°C (S4 Fig.). Beads coated with OmpA_G61A_, OmpA_K64A_, OmpA_GK6164AA_, and OmpA_KK6465AA_ were significantly compromised in their abilities to bind to and be internalized by HL-60 cells (Fig. 7, B and C). OmpA bead cellular adherence and entry were significantly inhibited and neutralized, respectively, for host cells that had been pretreated with α2,3/6-sialidase or α1,3/4-fucosidase (Fig. 7, D and E). Moreover, the sLe^x^-specific antibody, CSLEX1 significantly reduced binding and blocked internalization of OmpA beads into HL-60 cells (Fig. 7, F and G). KPL-1, an antibody that is specific for and blocks *A*. *phagocytophilum* binding to the PSGL-1 N-terminus [25,79,80], did not affect OmpA bead adherence or uptake (Fig. 7, H and I). These data indicate that OmpA coated beads bind and enter myeloid cells in a sLe^x^-dependent manner and require OmpA residues G61 and K64 to optimally do so.

### Delineation of the Asp14 binding domain

**Fig 8:**
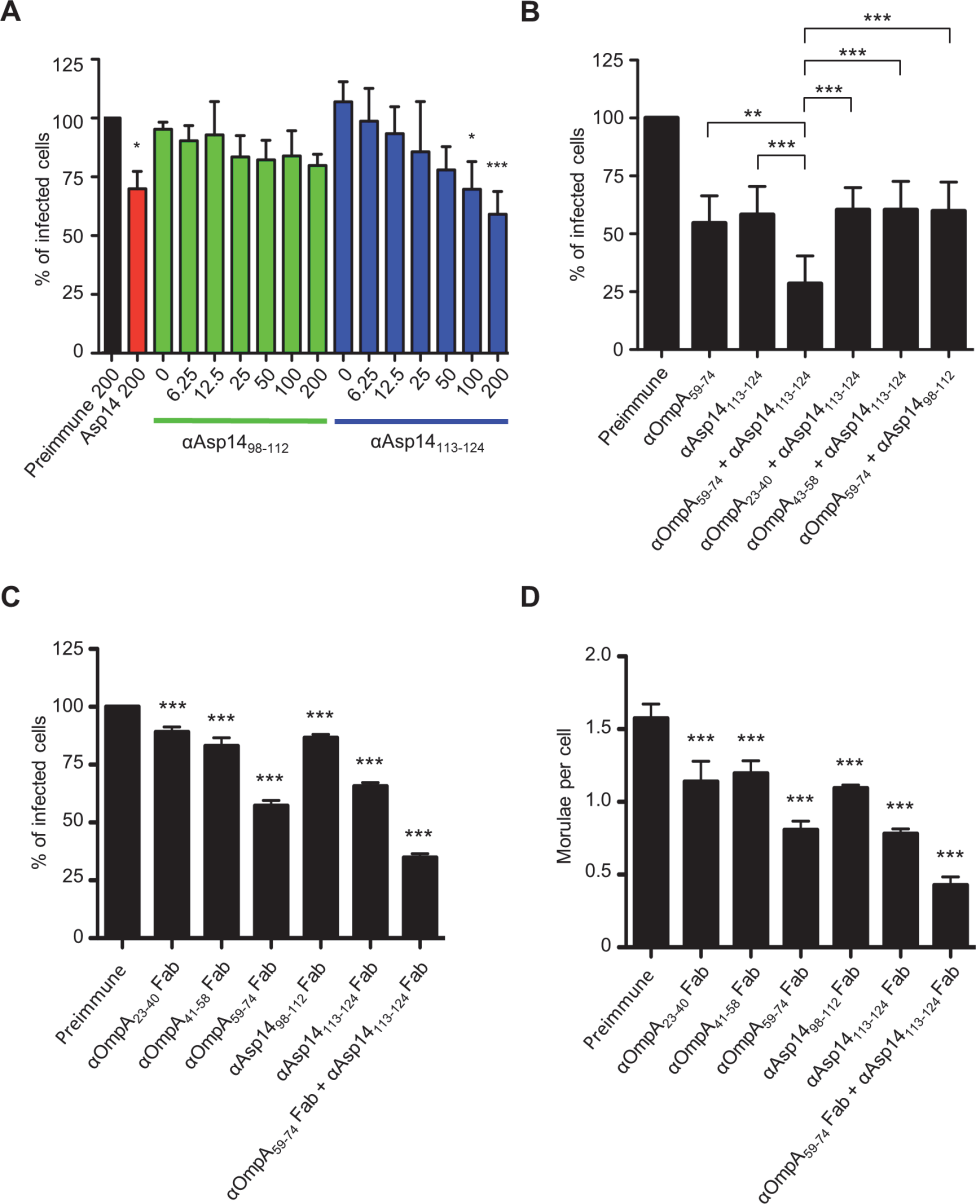
The Asp14 binding domain is contained within amino acids 113 to 124. (A) Pretreatment of *A*. *phagocytophilum* with Asp14_113–124_ antiserum inhibits infection of HL-60 cells in a dose-dependent manner. DC bacteria were incubated with 200 μg/ml of preimmune serum, 200 μg/ml of serum raised against full-length Asp14, or two-fold serially-diluted concentrations of anti-Asp14_98–112_ or anti-Asp14_113–124_ ranging from 0 to 200 μg/ml and then incubated with HL-60 cells. The infection was allowed to proceed for 24 h prior to being assessed by immunofluorescence microscopy for the percentage of infected cells. (B) A combination of antisera targeting OmpA_59–74_ and Asp14_113–124_ inhibits *A*. *phagocytophilum* infection of HL-60 cells better than serum targeting either binding domain alone. DC organisms were exposed to preimmune serum or antisera targeting OmpA_59–74_, Asp14_113–124_, OmpA_59–74_ plus Asp14_98–112_, or anti-Asp14_113–124_ together with OmpA_59–74_, OmpA_23–40_, or OmpA_43–58_ antibodies. The cells were fixed and screened using immunofluorescence microscopy to determine the percentages of infected cells. (C and D) OmpA_59–74_ and Asp14_113–124_ Fab fragments effectively inhibit *A*. *phagocytophilum* infection of HL-60 cells. DC bacteria were incubated with Fab fragments derived from preimmune serum, antibodies targeting OmpA_23–40_, OmpA_41–58_, OmpA_59–74_, Asp14_98–112_, Asp14_113–124_, or OmpA_59–74_ Fab fragment together with Asp14_113–124_ Fab fragment. The cells were fixed and screened to determine the percentages of infected cells (C) and morulae per cell (D). Results presented in (B) to (D) are relative to host cells that had been incubated with bacteria treated with preimmune serum. Results presented in (A) and (B) are the means ± SD for three experiments. Results in (C) and (D) are the mean ± SD of triplicate samples and are representative of two experiments with similar results. Statistically significant (* *P* < 0.05; ** *P* < 0.005; ***P < 0.001) values are indicated.

Of the three invasins that cooperatively function to facilitate *A*. *phagocytophilum* infection of mammalian host cells [19,29,36], only the binding domain of Asp14 had yet to be defined. Asp14 is a 124-amino acid (13.8 kDa) protein, and its binding domain lies within residues 101 to 124 [29]. To further narrow down this region, antisera were raised against residues 98 to 112 and 113 to 124. Both antisera recognized GST-Asp14, but not GST-Asp14_1–88_ or GST alone (S5 Fig.). Also, antiserum targeting Asp14_98–112_ but not Asp14_113–124_ detected GST-Asp14_1–112_ and each antiserum was specific for the peptide against which it had been raised. Next, the abilities of anti-Asp14_98–112_ and anti-Asp14_113–124_ to inhibit *A*. *phagocytophilum* infection of HL-60 cells were assessed. Incubating DC bacteria with Asp14_113–124_ antibody reduced the percentages of infected cells in a dose-dependent manner, whereas Asp14_98–112_ antibody had no effect (Fig. 8A). When used together, antisera against Asp14_113–124_ and OmpA_59–74_ reduced *A*. *phagocytophilum* by approximately four-fold (Fig. 8B). The observed blocking effect was significantly greater than that achieved with either antiserum alone or when either was paired with antisera that targeted irrelevant regions of OmpA or Asp14. To ensure that the blocking effects achieved by the OmpA_59–74_ and Asp14_113–124_ antisera were specific, fragment antigen binding (Fab fragment) portions of OmpA_23–40_, OmpA_41–48_, OmpA_59–74_, Asp14_98–112_, Asp14_113–124_, or OmpA_59–74_ and Asp14_113–124_ antibodies were prepared and assessed for the ability to inhibit *A*. *phagocytophilum* infection of HL-60 cells. Consistent with results obtained using intact antibodies, OmpA_59–74_ Fab, Asp14_113–124_ Fab, and the combination thereof achieved the greatest reductions in the percentage of infected cells and morulae per cell (Fig. 8, C and D).

### An antisera combination targeting the OmpA, Asp14, and AipA binding domains pronouncedly inhibits *A*. *phagocytophilum* infection of host cells

**Fig 9:**
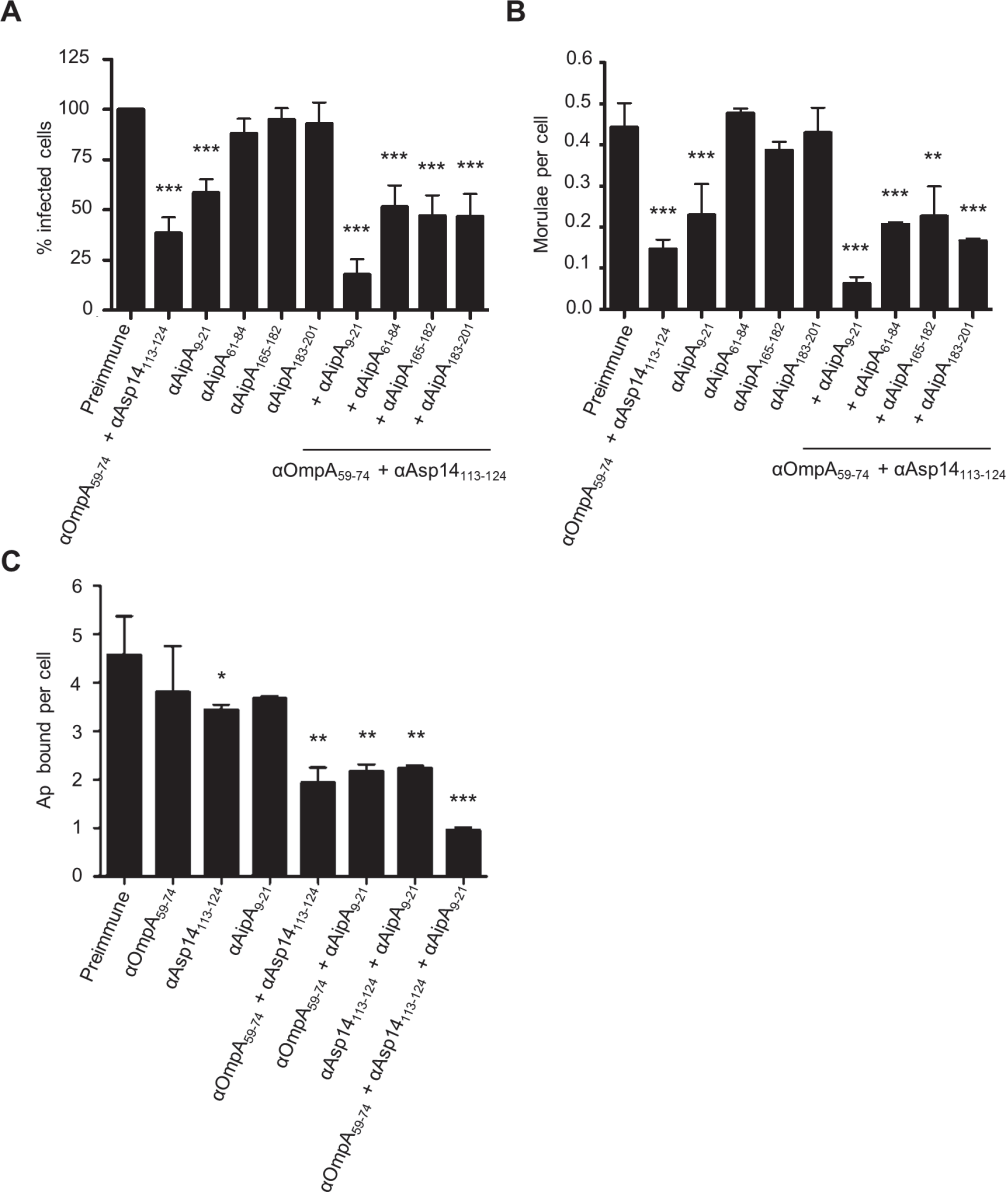
A combination of antisera targeting the binding domains of OmpA, Asp14, and AipA blocks *A*. *phagocytophilum* infection of mammalian host cells. DC organisms were incubated with preimmune serum or antibodies specific for OmpA_59–74_ and Asp14_113–124_; or AipA_9–21_, AipA_61–84_, AipA_165–182_, or AipA_183–201_, either independently or in combination with OmpA_59–74_ and Asp14_113–124_ antibodies. Next, the bacteria were incubated with HL-60 cells. The infection was allowed to proceed for 24 h, after which the host cells were fixed and examined using immunofluorescence microscopy to determine the percentages of infected cells (A) and the number of morulae per cell (B). (C) To verify that the observed reductions in *A*. *phagocytophilum* infection were due to antisera mediated blocking of bacterial binding to HL-60 cell surfaces, the experiment was repeated except that DC organisms were incubated with antibodies targeting OmpA_59–74_ and/or Asp14_113–124_, and/or AipA_9–21_ prior to being incubated with host cells, and the numbers of bound bacteria per cell was assessed. Results presented are relative to host cells that had been incubated with bacteria treated with preimmune serum and are the means ± SD for six combined experiments. Statistically significant (* *P* < 0.05; ** *P* < 0.005; ***P < 0.001) values are indicated.

We previously showed that a combination of antisera that had been raised against the entireties of OmpA, AipA, and Asp14 strongly inhibited *A*. *phagocytophilum* infection of mammalian host cells [36]. To refine this blocking approach, DC organisms were treated with a cocktail of antibodies specific for OmpA_59–74_, Asp14_113–124_, and AipA_9–21_ prior to incubating the bacteria with HL-60 cells. This antibody combination significantly attenuated infection, reducing the percentage of infected cells and number of morulae per cell by approximately five-fold (Fig. 9, A and B). The reduction in infection achieved using the combination antisera was due to effective blocking of bacterial adhesion to HL-60 cell surfaces, as combination antisera specific for OmpA_59–74_, Asp14_113–124_, and AipA_9–21_ reduced the numbers of bound *A*. *phagocytophilum* organisms per cell by more than four-fold relative to the same amount of preimmune serum (Fig. 9C). The observed reductions in bacterial adhesion and infection achieved by targeting all three binding domains were greater than those achieved using (1) antibodies that targeted only one or two of the binding domains and (2) combinations of antibodies against one or two of the binding domains together with antibodies against irrelevant portions of OmpA, Asp14, or AipA. Thus, targeting the OmpA, Asp14, and AipA binding domains together produced a synergistic blocking effect that protects host cells from *A*. *phagocytophilum* infection.

## Discussion

This study identified the OmpA and Asp14 binding domains and defined the OmpA residues that are critical for adhesion and invasion. The OmpA binding domain lies within amino acids 59 to 74 and it, like the rest of the protein, is highly conserved among *A*. *phagocytophilum* strains known to cause disease in humans and animals. Antibody against OmpA_59–74_ inhibited bacterial binding to PSGL-1 CHO cells and infection of HL-60 cells. OmpA_59–74_ is predicted to be a solvent exposed alpha helix and part of a cationic surface patch that binds sLe^x^, an interaction that is similar to those between staphylococcal superantigen-like (SSL) protein family members and sLe^x^. SSL4, SSL5, and SSL11 each use basic residues within cationic surface pockets to interact with α2,3-sialic acid of sLe^x^ [40,41,81]. Likewise, other pathogens’ sialic acid binding proteins, including uropathogenic *Escherichia coli* sialic acid-specific S fimbrial adhesin [82], pertussis toxin of *Bordetella pertussis* [43], influenza viral neuraminidase [44], canine adenovirus 2 capsid protein [83], and rhesus rotovirus VP4 [42] all use basic residues localized within cationic surface pockets to target sialic acid. The Asp14 binding domain is within amino acids 113 to 124. Antibody specific for Asp14_113–124_ abrogated bacterial binding and infection of host cells. As Asp14 bears no semblance to any known crystal structure, it could not be modeled. However, from the data presented herein it can be inferred that Asp14 amino acids 113 to 124 are exposed on the surfaces of *A*. *phagocytophilum* and the invasin itself.

OmpA K64 is essential for and G61 contributes to the ability of OmpA to bind to mammalian host cells. These experimental findings support the top two OmpA-sLe^x^ docking models, both of which predicted the involvement of K64 and G61 in interacting with α2,3-sialic acid and α1,3-fucose of sLe^x^. The actual interactions between OmpA and sLe^x^ are likely a hybrid of those predicted by the two docking models because, while both predicted the involvement of K64 and G61, one also predicted the involvement of K60, which was found to be negligible for OmpA to act as a competitive agonist. OmpA K64 and G61 may play functionally conserved roles among members of the family *Anaplasmataceae* and the genus *Anaplasma*. K64 is present in all *Anaplasma* and *Ehrlichia* spp. OmpA proteins, while G61 is conserved among *Anaplasma* but not *Ehrlichia* spp. OmpA proteins. *A*. *marginale* agglutinates bovine red blood cells in a sialidase-sensitive manner [84], indicating that it interacts with sialylated glycans on erythrocyte surfaces. Given the similarities between *A*. *phagocytophilum* and *A*. *marginale* OmpA proteins [19] and the conservation of residues implicated in receptor recognition, it will be worth investigating whether *A*. *marginale* OmpA is important for infection of bovine erythrocytes, and, if so, if it involves interactions between conserved OmpA lysine and glycine residues with sialylated glycans. *E*. *chaffeensis* OmpA contributes to infection of monocytic cells [85]. Compared to the conservation exhibited among *Anaplasma* spp. OmpA proteins, *A*. *phagocytophilum* and *E*. *chaffeensis* OmpA proteins are more divergent in sequence [19], especially in the binding domain, which may contribute to these pathogens’ tropisms for different leukocytes. Still, because of its conservation, the *E*. *chaffeensis* OmpA residue that corresponds to *A*. *phagocytophilum* OmpA K64 may be involved in binding to a sLe^x^-related glycan on monocytic cells.

Together with α2,3-sialic acid, α1,3-fucose is critical for *A*. *phagocytophilum* binding and infection [21,25–27]. OmpA binds α1,3-fucose, as can be inferred from our observations that recombinant OmpA bound poorly to RF/6A endothelial cells from which α1,3/4-fucose residues had been removed or that had been incubated with the α1,3/6-fucose-specific lectin, AAL. The ability of OmpA to bind α2,3-sialic acid and α1,3-fucose is consistent with the close proximity of the two sugar residues to each other in sLe^x^ and related glycans and also with OmpA-sLe^x^ molecular docking predictions. Yet, RF/6A cells, which support *A*. *phagocytophilum* binding and infection [19,28,29,31,33,34,36], express very little to no sLe^x^. Rather, they express 6-sulfo-sLe^x^, which presents α2,3-sialic acid and α1,3-fucose in the same orientation and proximity to each other as sLe^x^. Recombinant OmpA binding to RF/6A cells was significantly reduced in the presence of 6-sulfo-sLe^x^ antibody, but not sLe^x^ antibodies, thereby supporting that 6-sulfo-sLe^x^ is an *A*. *phagocytophilum* receptor on these cells. Thus, *A*. *phagocytophilum* OmpA interacts with glycans that present α2,3-sialic acid and α1,3-fucose in a similar manner as sLe^x^.

OmpA by itself functions as both an adhesin and an invasin, as demonstrated by the ability of His-OmpA to confer adhesive and internalization capabilities to inert beads. Approximately half of the His-OmpA beads that bound to host cells were internalized, a degree of uptake that was similar to that reported for *C*. *burnetii* OmpA coated beads [86]. Twenty-fold more OmpA coated beads bound to RF/6A cells than to HL-60 cells. Similarly, recombinant OmpA binding to RF/6A cells but not to HL-60 cells could be detected by immunofluorescence microscopy and flow cytometry. Nonetheless, the ability of recombinant OmpA to competitively antagonize *A*. *phagocytophilum* binding and infection of HL-60 cells demonstrates its ability to bind to the host cells, but it apparently does so at too low an avidity to remain bound during the wash steps associated with sample preparation for the detection methods used. The observed differences in OmpA binding to HL-60 versus RF/6A cells could be due to differences in the levels of sLe^x^ and 6-sulfo-sLe^x^ on HL-60 and RF/6A cell surfaces or perhaps due to the presence of an additional, undefined OmpA receptor on RF/6A cells. Yet another possibility is that the bacterium binds with a greater avidity to 6-sulfo-sLe^x^ than to sLe^x^.

Because of the essential and cooperative roles that OmpA, Asp14, and AipA play in the *A*. *phagocytophilum* lifecycle [19,26,27,29,36], blocking their ability to function can prevent both infection and bacterial survival. Moreover, directing the immune response to their binding domains could enhance protective efficacy. In this study, an antibody cocktail specific for the OmpA, Asp14, and AipA binding domains blocked *A*. *phagocytophilum* infection of host cells. This finding could potentially pave the way for development of a multi-invasin targeting vaccine that can protect against or treat human and veterinary granulocytic anaplasmosis. The relevance of this work extends to other obligate intracellular pathogens that use multiple invasins, including *A*. *marginale* [87], *E*. *chaffeensis* [85,88,89], spotted fever rickettsiae [90–94], *Chlamydia* spp. [95–99], *Mycobacterium* spp. [100–102], and *Orientia tsutsugamushi* [103,104], as their survival hinges on their abilities to enter host cells.

## Materials and Methods

### Cell lines and cultivation of *A*. *phagocytophilum*

Uninfected and *A*. *phagocytophilum* infected (NCH-1 strain) HL-60 cells (ATCC CCL-240) and RF/6A cells (ATCC CRL-1790, Manassas, VA) were maintained as previously described [18,28]. CHO (-) and PSGL-1 CHO cells were cultivated as described [105].

### Site directed mutagenesis and recombinant proteins

pGST-OmpA, which encodes OmpA_19–205_ N-terminally fused to GST, was previously constructed [19]. Using pGST-OmpA as template and primers from suppl. S1 Table, the QuikChange Lightning (Agilent Technologies, Santa Clara, CA) protocol was used per the manufacturer’s guidelines to perform site-directed insertions and point mutagenesis of the *ompA* insert sequence. For site directed insertions, a five-amino acid insert sequence (CLNHL) was selected based on previous studies that had successfully employed the linker-scanning method [58,60], which is used to insert peptide “linkers” to disrupt protein binding domains without perturbing overall protein structure. The sequence chosen for the insertion peptide, CLNHL, was a consensus sequence based on the most common amino acids at their respective positions in the insertion peptides used in prior studies [58,60]. The nucleotide sequence, 5'-TGCCTGAACCACCTG-3', which encoded CLNHL, was inserted in the *ompA* sequence of pGST-OmpA between *ompA* nucleotides 102 and 103, 162 and 163, 186 and 187, 201 and 202, 216 and 217, and 231 and 232 to yield plasmids that encoded GST-OmpA proteins bearing CLNHL inserts between OmpA amino acids 34 and 35, 54 and 55, 62 and 63, 67 and 68, 72 and 73, and 77 and 78, respectively. Likewise, the QuikChange protocol was used to perform site directed mutagenesis to yield plasmids that encoded GST-OmpA proteins having R32, D53, K60, G61, K64, K65, E69, and/or E72 converted to alanine. GST-OmpA mutants were expressed and purified as previously described [19]. Plasmids encoding His-tagged wild type and site-directed mutant OmpA proteins were generated by amplifying wild type and mutant *ompA* sequences using primers containing ligase-independent cloning (LIC) tails and annealing the amplicons into the pET46 Ek/LIC vector (Novagen, EMD Millipore, Darmstadt, DE) per the manufacturer’s instructions. His-OmpA proteins were expressed and purified by immobilized metal-affinity chromatography as previously described [106].

### Molecular modeling of the OmpA-sLe^x^ interaction

To obtain a putative three-dimensional OmpA protein structure, the mature OmpA sequence was threaded onto the solved crystal structures of proteins with similar sequences using the PHYRE2 server (www.sbg.bio.ic.ac.uk/phyre2/html/page.cgi) as previously described [19,38]. Amino acids 19 to 150 (73% of the mature OmpA sequence) were modeled with greater than 90% confidence to known structures for similar proteins (Protein Data Bank [PDB] files 2aiz [*Haemophilus influenzae* OmpP6 peptidoglycan associated lipoprotein (PAL)], 4g4v [*Acinetobacter baumanni* PAL], 4b5c [*Burkholderia pseudomallei* PAL], 3ldt [*Legionella pneumophila* OmpA], 2kgw [*Mycobaterium tuberculosis* OmpATb]). The remainder of the protein lacked sufficient homology to any experimentally derived structure, but could be modeled using the Poing method [38], which was performed as part of the Phyre2 analyses. The sLe^x^-PSGL-1 peptide (residues 61 to 77) and the sLe^x^ glycan itself was extracted from the solved crystal structure of PSGL-1 (PDB 1G1S) in PyMol (www.pymol.org) and saved as an individual PDB file. Open Babel software was used to convert PDB files to PDBQT (Protein Data Bank, Partial Charge and Atom Type) format in order to perform OmpA-sLe^x^ docking analysis [107]. AutoDock Tools software (autodock.scripps.edu/resources/adt) was used to generate the docking output files for both the OmpA protein structure and the sLe^x^ ligand. The search location for OmpA was generated in AutoDock Tools by setting a search grid that encompassed OmpA residues 19 to 74 [19]. Molecular docking was performed using AutoDock Vina (http://vina.scripps.edu/) to identify potential points of interaction between OmpA and sLe^x^ [45]. The top two OmpA-sLe^x^ models generated by AutoDock Vina had the same predicted affinity value of -4.2 kcal/mol and were selected for analysis in PyMol to determine potential points of contact.

### Antibodies, reagents, enzyme-linked immunosorbent assay (ELISA), and western blotting

To generate antisera specific to the OmpA and Asp14 binding domains, peptides corresponding to OmpA residues 23 to 40, 41 to 58, and 59 to 74 and Asp14 residues 98 to 112 and 113 to 124 were synthesized, conjugated to keyhole limpet hemocyanin, administered to rabbits, and the resulting OmpA and Asp14 peptide-specific sera were affinity-purified by New England Peptide (Gardner, MA). Each peptide antiserum’s specificity for the peptide against which it had been raised and for its protein target was determined by ELISA using the TMB substrate kit (Thermo Scientific, Waltham, MA) following the manufacturer’s instructions or by Western blot analysis as previously described [108]. Mouse anti-AipA peptide antisera have been previously described [36]. sLe^x^ antibodies CSLEX1 (BD Biosciences, San Jose, CA) and KM93 (Millipore, Darmstadt, DE) and PSGL-1 N-terminus-specific antibody KPL-1 (BD Biosciences) were obtained commercially. Fab fragments of OmpA and Asp14 peptide-specific antisera were generated using the Fab Preparation Kit (Pierce, Rockford, IL) according to the manufacturer’s instructions. Reiji Kannagi (Aichi Medical University, Nagukute, Aichi, Japan) kindly provided 6-sulfo-sLe^x^ antibody, G72. His tag and Alexa Fluor 488-conjugated secondary antibodies and Alexa Fluor 488-conjugated streptavidin were obtained from Invitrogen (Carlsbad, CA). Biotinylated AAL and MAL II were obtained from Vector Labs (Burlingame, CA). Glycosidases used in this study were α2,3/6-sialidase (Sigma-Aldrich, St. Louis, MO) and α1,3/4-fucosidase (Clontech, Mountain View, CA).

### Sequence alignments

The NCH-1 gene sequence for *ompA* (APH0338) was previously determined [19,29,36]. A Protein BLAST (basic local alignment search tool) [109] search using the NCH-1 OmpA predicted protein sequence as the query was used to identify homologs in other *Anaplasmataceae* species and in *A*. *phagocytophilum* strains HZ [50], HGE1 [54], Dog [53], JM [52], MRK [48,49], CRT35, CRT38 [51], and NorV2 [53], for which the genomes are available [53,110]. All of these strains except for NorV2 had been originally isolated from clinically affected humans and animals. HZ and HGE1 were recovered from human patients in Westchester, NY, USA and Minnesota, USA, respectively [50,54]. The Dog and JM strains were isolated from a dog in Minnesota, USA and a meadow jumping mouse (*Zapus hudsonius*) in Camp Ripley, MN, USA [52,53]. MRK had been recovered from a horse in California, USA [48,49]. CRT35 and CRT38 are isolates of the *A*. *phagocytophilum Ap*-variant 1 strain that were recovered from ticks collected at Camp Ripley, MN, USA [51]. NorV2 is a naturally occurring *A*. *phagocytophilum* isolate that was maintained in an experimentally infected lamb, exhibits reduced virulence in sheep, and differs in its 16S rRNA gene sequence when compared to other sheep isolates [53,111]. OmpA sequence alignments were generated using Clustal W [112].

### Binding of recombinant OmpA proteins to host cells

For binding of His- or GST-tagged OmpA proteins to host cells, RF/6A or HL-60 cells were incubated with 4 μM recombinant protein in culture media for 1 h in a 37^°^C incubator supplemented with 5% CO_2_ and a humidified atmosphere. To assess for the presence of sLe^x^ or 6-sulfo-sLe^x^ on RF/6A cell surfaces, the cells were fixed in 4% PFA in PBS for 1 h at room temperature followed by incubation with CSLEX1, KM93, or G72 for 1 h at room temperature. Antibody incubations and washes were performed as described previously [79]. Spinning-disk confocal microscopy using an Olympus BX51 microscope affixed with a disk-spinning unit (Olympus, Center Valley, PA) and/or flow cytometry using a BD FACS Canto II (BD Biosciences) were performed to assess binding of antibodies or His-OmpA proteins to host cell surfaces as previously described [19,29]. In some cases, RF/6A cells were pretreated with α2,3/6-sialidase, α1,3/4-fucosidase, AAL, MAL II, or sLe^x-^ or 6-sulfo-sLe^x^-specific antibodies prior to incubation with His-OmpA.

### Competitive inhibition of *A*. *phagocytophilum* binding and infection

Competitive inhibition assays utilizing recombinant protein or antibody were performed and analyzed by spinning-disk confocal microscopy as previously described [19,29]. To determine if *A*. *phagocytophilum* binding to PSGL-1 CHO cells or infection of RF/6A cells involved bacterial binding to host cell surface fucose residues, the host cells were treated with α1,3/4-fucosidase (10 μU/mL) prior to the addition of DC organisms and assessment for bacterial binding or infection as previously described [18,19]. For competitive inhibition assays using antisera raised against OmpA or Asp14 peptides, *A*. *phagocytophilum* DC bacteria were incubated with serially diluted concentrations of antiserum. Preimmune rabbit serum (200 μg/mL) was a negative control. Assays using combinations of two or three different OmpA, Asp14, or AipA peptide antibodies were performed using 100 μg/mL per antibody. Preimmune serum (200 μg/mL or 300 μg/mL, based on the combined total of peptide antisera) served as a negative control. Competitive inhibition assays using OmpA and/or Asp14 Fab fragments were performed exactly as described for antisera. Preimmune Fab fragments served as a negative control.

### OmpA coated bead uptake assay

1.8 x 10^7^ red fluorescent sulfate-modified 1.0 μm diameter microfluorospheres (Life Technologies, Carlsbad, CA) were mixed by rotation with 8 μg of His-OmpA, or His-OmpA proteins bearing alanine substitutions, in 400 μL of 50 mM phosphate-buffered saline (PBS) supplemented with 0.9% NaCl at room temperature overnight in the absence of light. The His-OmpA coated beads were centrifuged at 5,000 *g* for 25 min, followed by three washes in 50 mM PBS. Coated beads were resuspended in 400 μL of 50 mM PBS, 0.9% NaCl, 1% BSA and stored at 4^°^C until use. To validate that the beads were conjugated with His-OmpA, 1.8x10^4^ of the beads were screened by immunofluorescent microscopy using mouse polyclonal OmpA antisera followed by Alexa Fluor 488-conjugated goat anti-mouse IgG as described [19]. To assess binding to and uptake by HL-60 or RF/6A cells, His-OmpA coated beads or uncoated control beads were resuspended in the appropriate culture medium and added to host cells at a concentration of 500 beads/cell. For adherent RF/6A cells, beads were centrifuged onto the host cells at 1,000 *g* for 5 min. The cells plus beads were incubated for 1 h at 37^°^C in a 5% CO_2_ supplemented humidified incubator followed by washing the cells three times with PBS to remove unbound beads. Non-adherent HL-60 cells were mixed with the beads in suspension, incubated as described above, and three PBS washes were performed intermittently between five-min spins performed at 300 *g*. To assess binding, the host cells were fixed in 4% paraformaldehyde (PFA) in PBS, mounted with ProLong Antifade Gold gel mounting medium containing 4',6-diamidino-2-phenylindole (DAPI) (Invitrogen), and analyzed by spinning-disk confocal microscopy as previously described [19]. For uptake assays, after the final wash, the host cells were resuspended in culture medium and cultivated for an additional 7 h. The cells were washed three times in PBS, incubated with a 0.25% trypsin solution (Hyclone, Thermo Scientific, Waltham, MA) for 10 min at 37^°^C to cleave host cell surface proteins and consequently remove non-internalized beads, and washed three times with PBS. HL-60 cells were cytospun onto glass microscope slides and fixed, mounted, and screened as described above. RF/6A cells were added to wells containing coverslips, incubated overnight in a 37^°^C incubator supplemented with 5% CO_2_ and a humidified atmosphere to allow the host cells to adhere prior to further processing. To determine if His-OmpA coated bead binding or uptake was temperature sensitive, some experiments were performed at 4^°^C. To assess the contribution of sLe^x^ or PSGL-1 determinants to His-OmpA coated bead binding and uptake, host cells were pretreated with α2,3-sialidase (5 μg/mL), α1,3/4-fucosidase (10 μU/mL), sLe^x^-specific antibody CSLEX1 (10 μg/mL), PSGL-1 N-terminus-specific antibody KPL-1 (10 μg/mL), or vehicle or isotype controls as previously described [19] prior to the bead binding and uptake assays.

### Scanning electron microscopy

Coverslips of RF/6A cells were incubated with OmpA coated or control beads as described above. The coverslips were fixed in 2.0% glutaraldehyde in 0.1 M sodium cacodylate for 1 h at room temperature. The coverslips were subjected to two 10-min washes in 0.1 M sodium cadodylate and fixed in 1.0% osmium tetroxide in 0.1 M sodium cacodylate for 1 h. The coverslips were rinsed two more times with 0.1 M sodium cadodylate buffer for 10 min each. The samples were dehydrated by successive 5-min incubations in 50% ethanol, 70% ethanol, 80% ethanol, 95% ethanol, and three 10-min washes in 10% ethanol. Next, the samples were incubated three times for 30 min each in hexamethyldisilazane, air-dried, mounted with silver paint, and sputter coated with gold before imaging on a Zeiss EVO 50XVP scanning electron microscope (Thornwood, NY). For HL-60 cells incubated in suspension with beads, the samples were retained on a 0.1 μm filter and processed exactly as described for RF/6A cells.

### Statistical analyses

The Prism 5.0 software package (Graphpad, San Diego, CA) was used to determine the statistical significance of data using one-way analysis of variance (ANOVA) or the Student’s T-test, as previously described [19]. Statistical significance was set to *P* < 0.05.

## Supporting Information

S1 Fig Validation of OmpA peptide-specific antisera. Antibodies raised against peptides corresponding to OmpA_23–40_, OmpA_41–58_, and OmpA_59–74_ were used to screen Western-blotted GST-tagged OmpA, OmpA_19–74_, OmpA_75–205_, and GST alone (A) or Western-blotted His-OmpA or His-Asp14 (B) to confirm that each antibody was specific for the recombinant forms of OmpA that contained the target peptide sequences. (C) ELISA in which OmpA_23–40_, OmpA_41–58_, and OmpA_59–74_ antibodies were serially diluted two-fold from 1:200 to 1:409,600 and used to screen wells coated with GST, GST-OmpA, GST-OmpA_19–74_, GST-OmpA_75–205_, or peptides corresponding to OmpA_23–40_, OmpA_41–58_, or OmpA_59–74_. Results shown are representative of three independent experiments with similar results.
(TIF)

S2 Fig OmpA is highly conserved among *A*. *phagocytophilum* isolates and its key binding residues exhibit variable conservation among *Anaplasmataceae* species. (A) Alignment of OmpA amino acid sequence from the *A*. *phagocytophilum* NCH-1 strain (isolated from a human patient in Massachusetts), with OmpA sequences from *A*. *phagocytophilum* strains HZ (human; New York), HGE1 (human; Minnesota), Dog (Minnesota), JM (jumping mouse; Minnesota), MRK (horse; California), *Ap*Var-1 isolates CRT35 and CRT38 (both from ticks; Minnesota), and NorV2 (lamb; Norway). (B) Alignment of NCH-1 OmpA amino acids 19 to 74 with corresponding regions of OmpA homologs from the *A*. *marginale* St. Maries strain (AM854), *A*. *marginale* Florida strain (AMF640), *A*. *marginale* subsp. *centrale* Israel starin (ACIS00486) *E*. *chaffeensis* Arkansas strain (ECH0462), *Ehrlichia canis* Jake strain (Ecaj0563), and the *Ehrlichia ruminantium* Welgevonden strain (Erum5620). The binding domain corresponding to NCH-1 OmpA residues 59 to 74 is highlighted with blue in (A) and (B). Red text in (A) and (B) denotes amino acids that were mutated to alanine for the experiments presented in Fig. 3 panels B to D. Numbers above the alignments in (A) and (B) denote amino acid position numbers. The arrows in (A) and (B) denote *A*. *phagocytophilum* OmpA G61 and K64, which were predicted to form interactions with sLe^x^ in Fig. 2 panels D and E and were shown to be critical for OmpA to bind to and mediate infection of mammalian host cells in Figs. 1 and 3.
(TIF)

S3 Fig Treatment with α1,3/4-fucosidase reduces *A*. *phagocytophilum* binding to PSGL-1 CHO cells and binding to and infection of RF/6A endothelial cells. PSGL-1 CHO cells (A) and RF/6A cells (B and C) were treated with α1,3/4-fucosidase (+ fucosidase) or vehicle control (- fucosidase). Fucosidase- and mock-treated cells were incubated with *A*. *phagocytophilum* DC organisms. Following the removal of unbound bacteria, the infection of RF/6A cells was allowed to proceed for 24 h prior to being assessed, while bacterial binding to PSGL-1 CHO and RF/6A cells was examined immediately. The mean number (± SD) of bound DC bacteria per PSGL-1 CHO (A) or RF/6A cell (B) or percentage of infected RF/6A cells (C) were determined using immunofluorescence microscopy. Results shown are the means ± SD for three combined experiments. Statistically significant (***P < 0.001) values are indicated.
(TIF)

S4 Fig OmpA coated bead uptake by promyelocytic HL-60 cells is inhibited at 4°C. HL-60 cells were incubated with OmpA coated beads or non-coated control beads at 37°C or 4°C. The mean numbers (± SD) of bound (A) and internalized beads (B) were determined using immunofluorescence microscopy. Results presented are representative of three experiments performed in triplicate with similar results. Statistically significant (***P < 0.001) values are indicated.
(TIF)

S5 Fig Validation of Asp14 peptide-specific antisera. Antibodies raised against peptides corresponding to Asp14_98–112_ or Asp14_113–124_ were used to screen Western-blotted GST-Asp14, GST-Asp14_1–88_, GST-Asp14_1–112_, and GST alone (A) or Western-blotted His-Asp14 or His-OmpA (B) to confirm that each antibody was specific for the Asp14 target peptide sequences. (C) ELISA in which serially diluted antibodies raised against Asp14_98–112_ and Asp14_113–124_ were used to screen wells coated with GST, GST-Asp14, GST-Asp14_1–112_, GST-Asp14_1–88_, or peptides corresponding to Asp14_98–112_ or Asp14_113–124_. Results shown are representative of three independent experiments with similar results.
(TIF)

S1 Table OmpA oligonucleotides used in this study. (DOCX)

## References

[1] Truchan HK, Seidman D, Carlyon JA (2013) Breaking in and grabbing a meal: Anaplasma phagocytophilum cellular invasion, nutrient acquisition, and promising tools for their study. Microbes Infect. 10.1016/j.micinf.2013.10.010 PMC3894830 24141091

[2] CDC (2013) Notice to readers: final 2012 reports of nationally notifiable infectious diseases. MMWR Morb Mortal Wkly Rep 62: 669–682. 24133698 PMC4604800

[3] Hopkins RS, Jajosky RA, Hall PA, Adams DA, Connor FJ, et al (2005) Summary of notifiable diseases—United States, 2003. MMWR Morb Mortal Wkly Rep 52: 1–85. 15889005

[4] Hao Q, Geng Z, Hou XX, Tian Z, Yang XJ, et al (2013) Seroepidemiological investigation of lyme disease and human granulocytic anaplasmosis among people living in forest areas of eight provinces in China. Biomed Environ Sci 26: 185–189. 10.3967/0895-3988.2013.03.005 23425801

[5] Zhang XC, Zhang LX, Li WH, Wang SW, Sun YL, et al (2012) Ehrlichiosis and zoonotic anaplasmosis in suburban areas of Beijing, China. Vector Borne Zoonotic Dis 12: 932–937. 10.1089/vbz.2012.0961 23025695

[6] Zhang S, Hai R, Li W, Li G, Lin G, et al (2009) Seroprevalence of human granulocytotropic anaplasmosis in central and southeastern China. Am J Trop Med Hyg 81: 293–295. 19635886

[7] Aguero-Rosenfeld ME, Donnarumma L, Zentmaier L, Jacob J, Frey M, et al (2002) Seroprevalence of antibodies that react with Anaplasma phagocytophila, the agent of human granulocytic ehrlichiosis, in different populations in Westchester County, New York. J Clin Microbiol 40: 2612–2615. 12089287 10.1128/JCM.40.7.2612-2615.2002 PMC120546

[8] Bakken JS, Goellner P, Van Etten M, Boyle DZ, Swonger OL, et al (1998) Seroprevalence of human granulocytic ehrlichiosis among permanent residents of northwestern Wisconsin. Clin Infect Dis 27: 1491–1496. 9868666 10.1086/515048

[9] Alhumaidan H, Westley B, Esteva C, Berardi V, Young C, et al (2013) Transfusion-transmitted anaplasmosis from leukoreduced red blood cells. Transfusion 53: 181–186. 10.1111/j.1537-2995.2012.03685.x 22563784

[10] Annen K, Friedman K, Eshoa C, Horowitz M, Gottschall J, et al (2012) Two cases of transfusion-transmitted Anaplasma phagocytophilum. Am J Clin Pathol 137: 562–565. 10.1309/AJCP4E4VQQQOZIAQ 22431531

[11] CDC (2008) Anaplasma phagocytophilum transmitted through blood transfusion-Minnesota. MMWR Morb Mortal Wkly Rep 57: 1145–1148. 18946461

[12] Dhand A, Nadelman RB, Aguero-Rosenfeld M, Haddad FA, Stokes DP, et al (2007) Human granulocytic anaplasmosis during pregnancy: case series and literature review. Clin Infect Dis 45: 589–593. 17682993 10.1086/520659

[13] Jereb M, Pecaver B, Tomazic J, Muzlovic I, Avsic-Zupanc T, et al (2012) Severe human granulocytic anaplasmosis transmitted by blood transfusion. Emerg Infect Dis 18: 1354–1357. 10.3201/eid1808.120180 22841007 PMC3414041

[14] Stuen S, Granquist EG, Silaghi C (2013) Anaplasma phagocytophilum—a widespread multi-host pathogen with highly adaptive strategies. Front Cell Infect Microbiol 3: 31 10.3389/fcimb.2013.00031 23885337 PMC3717505

[15] Bastidas RJ, Elwell CA, Engel JN, Valdivia RH (2013) Chlamydial intracellular survival strategies. Cold Spring Harb Perspect Med 3: a010256 10.1101/cshperspect.a010256 23637308 PMC3633179

[16] Minnick MF, Raghavan R (2012) Developmental biology of Coxiella burnetii. Adv Exp Med Biol 984: 231–248. 10.1007/978-94-007-4315-1_12 22711635

[17] Zhang JZ, Popov VL, Gao S, Walker DH, Yu XJ (2007) The developmental cycle of Ehrlichia chaffeensis in vertebrate cells. Cell Microbiol 9: 610–618. 16987329 10.1111/j.1462-5822.2006.00812.x

[18] Troese MJ, Carlyon JA (2009) Anaplasma phagocytophilum dense-cored organisms mediate cellular adherence through recognition of human P-selectin glycoprotein ligand 1. Infect Immun 77: 4018–4027. 10.1128/IAI.00527-09 19596771 PMC2738047

[19] Ojogun N, Kahlon A, Ragland SA, Troese MJ, Mastronunzio JE, et al (2012) Anaplasma phagocytophilum outer membrane protein A interacts with sialylated glycoproteins to promote infection of mammalian host cells. Infect Immun 80: 3748–3760. 10.1128/IAI.00654-12 22907813 PMC3486060

[20] Sperandio M (2006) Selectins and glycosyltransferases in leukocyte rolling in vivo. FEBS J 273: 4377–4389. 16956372 10.1111/j.1742-4658.2006.05437.x

[21] Goodman JL, Nelson CM, Klein MB, Hayes SF, Weston BW (1999) Leukocyte infection by the granulocytic ehrlichiosis agent is linked to expression of a selectin ligand. J Clin Invest 103: 407–412. 9927502 10.1172/JCI4230 PMC407896

[22] Karakantza M, Gibson FM, Cavenagh JD, Ball SE, Gordon MY, et al (1994) SLe(x) expression of normal CD34 positive bone marrow haemopoietic progenitor cells. Br J Haematol 86: 883–886. 7522525 10.1111/j.1365-2141.1994.tb04850.x

[23] Symington FW, Hedges DL, Hakomori S (1985) Glycolipid antigens of human polymorphonuclear neutrophils and the inducible HL-60 myeloid leukemia line. J Immunol 134: 2498–2506. 3855933

[24] Fukuda M, Spooncer E, Oates JE, Dell A, Klock JC (1984) Structure of sialylated fucosyl lactosaminoglycan isolated from human granulocytes. J Biol Chem 259: 10925–10935. 6432790

[25] Herron MJ, Nelson CM, Larson J, Snapp KR, Kansas GS, et al (2000) Intracellular parasitism by the human granulocytic ehrlichiosis bacterium through the P-selectin ligand, PSGL-1. Science 288: 1653–1656. 10834846 10.1126/science.288.5471.1653

[26] Carlyon JA, Akkoyunlu M, Xia L, Yago T, Wang T, et al (2003) Murine neutrophils require alpha1,3-fucosylation but not PSGL-1 for productive infection with Anaplasma phagocytophilum. Blood 102: 3387–3395. 12869507 10.1182/blood-2003-02-0621

[27] Yago T, Leppanen A, Carlyon JA, Akkoyunlu M, Karmakar S, et al (2003) Structurally distinct requirements for binding of P-selectin glycoprotein ligand-1 and sialyl Lewis x to Anaplasma phagocytophilum and P-selectin. J Biol Chem 278: 37987–37997. 12847092 10.1074/jbc.M305778200

[28] Huang B, Ojogun N, Ragland SA, Carlyon JA (2012) Monoubiquitinated proteins decorate the Anaplasma phagocytophilum-occupied vacuolar membrane. FEMS Immunol Med Microbiol 64: 32–41. 10.1111/j.1574-695X.2011.00873.x 22066989

[29] Kahlon A, Ojogun N, Ragland SA, Seidman D, Troese MJ, et al (2013) Anaplasma phagocytophilum Asp14 is an invasin that interacts with mammalian host cells via its C terminus to facilitate infection. Infect Immun 81: 65–79. 10.1128/IAI.00932-12 23071137 PMC3536139

[30] Mastronunzio JE, Kurscheid S, Fikrig E (2012) Postgenomic analyses reveal development of infectious Anaplasma phagocytophilum during transmission from ticks to mice. J Bacteriol 194: 2238–2247. 10.1128/JB.06791-11 22389475 PMC3347074

[31] Munderloh UG, Lynch MJ, Herron MJ, Palmer AT, Kurtti TJ, et al (2004) Infection of endothelial cells with Anaplasma marginale and A. phagocytophilum. Vet Microbiol 101: 53–64. 15201033 10.1016/j.vetmic.2004.02.011

[32] Schaff UY, Trott KA, Chase S, Tam K, Johns JL, et al (2010) Neutrophils exposed to A. phagocytophilum under shear stress fail to fully activate, polarize, and transmigrate across inflamed endothelium. Am J Physiol Cell Physiol 299: C87–96. 10.1152/ajpcell.00165.2009 20392928 PMC2904253

[33] Sukumaran B, Mastronunzio JE, Narasimhan S, Fankhauser S, Uchil PD, et al (2011) Anaplasma phagocytophilum AptA modulates Erk1/2 signalling. Cell Microbiol 13: 47–61. 10.1111/j.1462-5822.2010.01516.x 20716207 PMC3005019

[34] Xiong Q, Rikihisa Y (2011) The prenylation inhibitor manumycin A reduces the viability of Anaplasma phagocytophilum. J Med Microbiol 60: 744–749. 10.1099/jmm.0.029231-0 21349982 PMC3167922

[35] Ojogun N, Barnstein B, Huang B, Oskeritzian CA, Homeister JW, et al (2011) Anaplasma phagocytophilum infects mast cells via alpha1,3-fucosylated but not sialylated glycans and inhibits IgE-mediated cytokine production and histamine release. Infect Immun 79: 2717–2726. 10.1128/IAI.00181-11 21536789 PMC3191951

[36] Seidman D, Ojogun N, Walker NJ, Mastronunzio J, Kahlon A, et al (2014) Anaplasma phagocytophilum surface protein AipA mediates invasion of mammalian host cells. Cell Microbiol 16: 1133–1145. 10.1111/cmi.12286 24612118 PMC4115035

[37] Xia L, Ramachandran V, McDaniel JM, Nguyen KN, Cummings RD, et al (2003) N-terminal residues in murine P-selectin glycoprotein ligand-1 required for binding to murine P-selectin. Blood 101: 552–559. 12393631 10.1182/blood-2001-11-0036

[38] Kelley LA, Sternberg MJ (2009) Protein structure prediction on the Web: a case study using the Phyre server. Nat Protoc 4: 363–371. 10.1038/nprot.2009.2 19247286

[39] Baker NA, Sept D, Joseph S, Holst MJ, McCammon JA (2001) Electrostatics of nanosystems: application to microtubules and the ribosome. Proc Natl Acad Sci U S A 98: 10037–10041. 11517324 10.1073/pnas.181342398 PMC56910

[40] Chung MC, Wines BD, Baker H, Langley RJ, Baker EN, et al (2007) The crystal structure of staphylococcal superantigen-like protein 11 in complex with sialyl Lewis X reveals the mechanism for cell binding and immune inhibition. Mol Microbiol 66: 1342–1355. 18045383 10.1111/j.1365-2958.2007.05989.x

[41] Hermans SJ, Baker HM, Sequeira RP, Langley RJ, Baker EN, et al (2012) Structural and functional properties of staphylococcal superantigen-like protein 4. Infect Immun 80: 4004–4013. 10.1128/IAI.00764-12 22949551 PMC3486064

[42] Dormitzer PR, Sun ZY, Wagner G, Harrison SC (2002) The rhesus rotavirus VP4 sialic acid binding domain has a galectin fold with a novel carbohydrate binding site. EMBO J 21: 885–897. 11867517 10.1093/emboj/21.5.885 PMC125907

[43] Stein PE, Boodhoo A, Armstrong GD, Heerze LD, Cockle SA, et al (1994) Structure of a pertussis toxin-sugar complex as a model for receptor binding. Nat Struct Biol 1: 591–596. 7634099 10.1038/nsb0994-591

[44] Varghese JN, McKimm-Breschkin JL, Caldwell JB, Kortt AA, Colman PM (1992) The structure of the complex between influenza virus neuraminidase and sialic acid, the viral receptor. Proteins 14: 327–332. 1438172 10.1002/prot.340140302

[45] Trott O, Olson AJ (2010) AutoDock Vina: improving the speed and accuracy of docking with a new scoring function, efficient optimization, and multithreading. J Comput Chem 31: 455–461. 10.1002/jcc.21334 19499576 PMC3041641

[46] Somers WS, Tang J, Shaw GD, Camphausen RT (2000) Insights into the molecular basis of leukocyte tethering and rolling revealed by structures of P- and E-selectin bound to SLe(X) and PSGL-1. Cell 103: 467–479. 11081633 10.1016/s0092-8674(00)00138-0

[47] Kolbert CP, Bruinsma ES, Abdulkarim AS, Hofmeister EK, Tompkins RB, et al (1997) Characterization of an immunoreactive protein from the agent of human granulocytic ehrlichiosis. J Clin Microbiol 35: 1172–1178. 9114402 10.1128/jcm.35.5.1172-1178.1997 PMC232724

[48] Madigan JE, Gribble D (1987) Equine ehrlichiosis in northern California: 49 cases (1968–1981). J Am Vet Med Assoc 190: 445–448. 3558086

[49] Gribble DH (1969) Equine ehrlichiosis. J Am Vet Med Assoc 155: 462–469. 5819585

[50] Rikihisa Y, Zhi N, Wormser GP, Wen B, Horowitz HW, et al (1997) Ultrastructural and antigenic characterization of a granulocytic ehrlichiosis agent directly isolated and stably cultivated from a patient in New York state. J Infect Dis 175: 210–213. 8985223 10.1093/infdis/175.1.210

[51] Massung RF, Levin ML, Munderloh UG, Silverman DJ, Lynch MJ, et al (2007) Isolation and propagation of the Ap-Variant 1 strain of Anaplasma phagocytophilum in a tick cell line. J Clin Microbiol 45: 2138–2143. 17475757 10.1128/JCM.00478-07 PMC1932999

[52] Johnson RC, Kodner C, Jarnefeld J, Eck DK, Xu Y (2011) Agents of human anaplasmosis and Lyme disease at Camp Ripley, Minnesota. Vector Borne Zoonotic Dis 11: 1529–1534. 10.1089/vbz.2011.0633 21867420 PMC3231789

[53] Al-Khedery B, Lundgren AM, Stuen S, Granquist EG, Munderloh UG, et al (2012) Structure of the type IV secretion system in different strains of Anaplasma phagocytophilum. BMC Genomics 13: 678 10.1186/1471-2164-13-678 23190684 PMC3556328

[54] Goodman JL, Nelson C, Vitale B, Madigan JE, Dumler JS, et al (1996) Direct cultivation of the causative agent of human granulocytic ehrlichiosis. N Engl J Med 334: 209–215. 8531996 10.1056/NEJM199601253340401

[55] Carlyon JA (2012) Establishing intracellular infection: modulation of host cell functions (Anaplasmataceae). In: Palmer GH, Azad A, editors. Intracellular Pathogens II: Rickettsiales. Washington, D. C.: ASM Press.

[56] Mansueto P, Vitale G, Cascio A, Seidita A, Pepe I, et al (2012) New insight into immunity and immunopathology of Rickettsial diseases. Clin Dev Immunol 2012: 967852 10.1155/2012/967852 21912565 PMC3170826

[57] Suarez CE, Noh S (2011) Emerging perspectives in the research of bovine babesiosis and anaplasmosis. Vet Parasitol 180: 109–125. 10.1016/j.vetpar.2011.05.032 21684084

[58] Anton BP, Raleigh EA (2004) Transposon-mediated linker insertion scanning mutagenesis of the Escherichia coli McrA endonuclease. J Bacteriol 186: 5699–5707. 15317774 10.1128/JB.186.17.5699-5707.2004 PMC516834

[59] Grande KK, Gustin JK, Kessler E, Ohman DE (2007) Identification of critical residues in the propeptide of LasA protease of Pseudomonas aeruginosa involved in the formation of a stable mature protease. J Bacteriol 189: 3960–3968. 17351039 10.1128/JB.01828-06 PMC1913401

[60] Okoye ME, Sexton GL, Huang E, McCaffery JM, Desai P (2006) Functional analysis of the triplex proteins (VP19C and VP23) of herpes simplex virus type 1. J Virol 80: 929–940. 16378995 10.1128/JVI.80.2.929-940.2006 PMC1346874

[61] Yamashita K, Kochibe N, Ohkura T, Ueda I, Kobata A (1985) Fractionation of L-fucose-containing oligosaccharides on immobilized Aleuria aurantia lectin. J Biol Chem 260: 4688–4693. 3988732

[62] Chandrasekaran EV, Chawda R, Rhodes JM, Locke RD, Piskorz CF, et al (2003) The binding characteristics and utilization of Aleuria aurantia, Lens culinaris and few other lectins in the elucidation of fucosyltransferase activities resembling cloned FT VI and apparently unique to colon cancer cells. Carbohydr Res 338: 887–901. 12681913 10.1016/s0008-6215(03)00021-1

[63] Wang WC, Cummings RD (1988) The immobilized leukoagglutinin from the seeds of Maackia amurensis binds with high affinity to complex-type Asn-linked oligosaccharides containing terminal sialic acid-linked alpha-2,3 to penultimate galactose residues. J Biol Chem 263: 4576–4585. 3350806

[64] Akahori T, Yuzawa Y, Nishikawa K, Tamatani T, Kannagi R, et al (1997) Role of a sialyl Lewis(x)-like epitope selectively expressed on vascular endothelial cells in local skin inflammation of the rat. J Immunol 158: 5384–5392. 9164959

[65] Izawa M, Kumamoto K, Mitsuoka C, Kanamori C, Kanamori A, et al (2000) Expression of sialyl 6-sulfo Lewis X is inversely correlated with conventional sialyl Lewis X expression in human colorectal cancer. Cancer Res 60: 1410–1416. 10728707

[66] Majuri ML, Rabina J, Niittymaki J, Tiisala S, Mattila P, et al (1999) High endothelial cells synthesize and degrade sLex. Putative implications for L-selectin-dependent recognition. FEBS Lett 455: 97–100. 10428480 10.1016/s0014-5793(99)00834-0

[67] Mitsuoka C, Kawakami-Kimura N, Kasugai-Sawada M, Hiraiwa N, Toda K, et al (1997) Sulfated sialyl Lewis X, the putative L-selectin ligand, detected on endothelial cells of high endothelial venules by a distinct set of anti-sialyl Lewis X antibodies. Biochem Biophys Res Commun 230: 546–551. 9015359 10.1006/bbrc.1996.6012

[68] Mitsuoka C, Sawada-Kasugai M, Ando-Furui K, Izawa M, Nakanishi H, et al (1998) Identification of a major carbohydrate capping group of the L-selectin ligand on high endothelial venules in human lymph nodes as 6-sulfo sialyl Lewis X. J Biol Chem 273: 11225–11233. 9556613 10.1074/jbc.273.18.11225

[69] Renkonen R, Mattila P, Majuri ML, Rabina J, Toppila S, et al (1997) In vitro experimental studies of sialyl Lewis x and sialyl Lewis a on endothelial and carcinoma cells: crucial glycans on selectin ligands. Glycoconj J 14: 593–600. 9298692 10.1023/a:1018536509950

[70] Sawada M, Takada A, Ohwaki I, Takahashi N, Tateno H, et al (1993) Specific expression of a complex sialyl Lewis X antigen on high endothelial venules of human lymph nodes: possible candidate for L-selectin ligand. Biochem Biophys Res Commun 193: 337–347. 7684905 10.1006/bbrc.1993.1629

[71] Paavonen T, Renkonen R (1992) Selective expression of sialyl-Lewis x and Lewis a epitopes, putative ligands for L-selectin, on peripheral lymph-node high endothelial venules. Am J Pathol 141: 1259–1264. 1281614 PMC1886755

[72] Majuri ML, Pinola M, Niemela R, Tiisala S, Natunen J, et al (1994) Alpha 2,3-sialyl and alpha 1,3-fucosyltransferase-dependent synthesis of sialyl Lewis x, an essential oligosaccharide present on L-selectin counterreceptors, in cultured endothelial cells. Eur J Immunol 24: 3205–3210. 7528675 10.1002/eji.1830241244

[73] Munro JM, Lo SK, Corless C, Robertson MJ, Lee NC, et al (1992) Expression of sialyl-Lewis X, an E-selectin ligand, in inflammation, immune processes, and lymphoid tissues. Am J Pathol 141: 1397–1408. 1281620 PMC1886750

[74] Ishibashi Y, Inouye Y, Okano T, Taniguchi A (2005) Regulation of sialyl-Lewis x epitope expression by TNF-alpha and EGF in an airway carcinoma cell line. Glycoconj J 22: 53–62. 15864435 10.1007/s10719-005-0292-7

[75] Toppila S, Paavonen T, Laitinen A, Laitinen LA, Renkonen R (2000) Endothelial sulfated sialyl Lewis x glycans, putative L-selectin ligands, are preferentially expressed in bronchial asthma but not in other chronic inflammatory lung diseases. Am J Respir Cell Mol Biol 23: 492–498. 11017914 10.1165/ajrcmb.23.4.4113

[76] Turunen JP, Majuri ML, Seppo A, Tiisala S, Paavonen T, et al (1995) De novo expression of endothelial sialyl Lewis(a) and sialyl Lewis(x) during cardiac transplant rejection: superior capacity of a tetravalent sialyl Lewis(x) oligosaccharide in inhibiting L-selectin-dependent lymphocyte adhesion. J Exp Med 182: 1133–1141. 7561686 10.1084/jem.182.4.1133 PMC2192292

[77] Fukushima K, Hirota M, Terasaki PI, Wakisaka A, Togashi H, et al (1984) Characterization of sialosylated Lewisx as a new tumor-associated antigen. Cancer Res 44: 5279–5285. 6386148

[78] Dohi T, Nemoto T, Ohta S, Shitara K, Hanai N, et al (1993) Different binding properties of three monoclonal antibodies to sialyl Le(x) glycolipids in a gastric cancer cell line and normal stomach tissue. Anticancer Res 13: 1277–1282. 8239497

[79] Reneer DV, Kearns SA, Yago T, Sims J, Cummings RD, et al (2006) Characterization of a sialic acid- and P-selectin glycoprotein ligand-1-independent adhesin activity in the granulocytotropic bacterium Anaplasma phagocytophilum. Cellular microbiology 8: 1972–1984. 16869829 10.1111/j.1462-5822.2006.00764.x

[80] Snapp KR, Ding H, Atkins K, Warnke R, Luscinskas FW, et al (1998) A novel P-selectin glycoprotein ligand-1 monoclonal antibody recognizes an epitope within the tyrosine sulfate motif of human PSGL-1 and blocks recognition of both P- and L-selectin. Blood 91: 154–164. 9414280

[81] Baker HM, Basu I, Chung MC, Caradoc-Davies T, Fraser JD, et al (2007) Crystal structures of the staphylococcal toxin SSL5 in complex with sialyl Lewis X reveal a conserved binding site that shares common features with viral and bacterial sialic acid binding proteins. J Mol Biol 374: 1298–1308. 17996251 10.1016/j.jmb.2007.09.091

[82] Morschhauser J, Hoschutzky H, Jann K, Hacker J (1990) Functional analysis of the sialic acid-binding adhesin SfaS of pathogenic Escherichia coli by site-specific mutagenesis. Infect Immun 58: 2133–2138. 2194961 10.1128/iai.58.7.2133-2138.1990 PMC258787

[83] Rademacher C, Bru T, McBride R, Robison E, Nycholat CM, et al (2012) A Siglec-like sialic-acid-binding motif revealed in an adenovirus capsid protein. Glycobiology 22: 1086–1091. 10.1093/glycob/cws073 22522600 PMC3382346

[84] McGarey DJ, Allred DR (1994) Characterization of hemagglutinating components on the Anaplasma marginale initial body surface and identification of possible adhesins. Infect Immun 62: 4587–4593. 7927725 10.1128/iai.62.10.4587-4593.1994 PMC303147

[85] Cheng Z, Miura K, Popov VL, Kumagai Y, Rikihisa Y (2011) Insights into the CtrA regulon in development of stress resistance in obligatory intracellular pathogen Ehrlichia chaffeensis. Mol Microbiol 82: 1217–1234. 10.1111/j.1365-2958.2011.07885.x 22014113 PMC3241975

[86] Martinez E, Cantet F, Fava L, Norville I, Bonazzi M (2014) Identification of OmpA, a Coxiella burnetii Protein Involved in Host Cell Invasion, by Multi-Phenotypic High-Content Screening. PLoS Pathog 10: e1004013 10.1371/journal.ppat.1004013 24651569 PMC3961360

[87] de la Fuente J, Garcia-Garcia JC, Blouin EF, Kocan KM (2001) Differential adhesion of major surface proteins 1a and 1b of the ehrlichial cattle pathogen Anaplasma marginale to bovine erythrocytes and tick cells. Int J Parasitol 31: 145–153. 11239934 10.1016/s0020-7519(00)00162-4

[88] Mohan Kumar D, Yamaguchi M, Miura K, Lin M, Los M, et al (2013) Ehrlichia chaffeensis uses its surface protein EtpE to bind GPI-anchored protein DNase X and trigger entry into mammalian cells. PLoS Pathog 9: e1003666 10.1371/journal.ppat.1003666 24098122 PMC3789761

[89] Popov VL, Yu X, Walker DH (2000) The 120 kDa outer membrane protein of Ehrlichia chaffeensis: preferential expression on dense-core cells and gene expression in Escherichia coli associated with attachment and entry. Microb Pathog 28: 71–80. 10644493 10.1006/mpat.1999.0327

[90] Cardwell MM, Martinez JJ (2009) The Sca2 autotransporter protein from Rickettsia conorii is sufficient to mediate adherence to and invasion of cultured mammalian cells. Infect Immun 77: 5272–5280. 10.1128/IAI.00201-09 19805531 PMC2786473

[91] Chan YG, Cardwell MM, Hermanas TM, Uchiyama T, Martinez JJ (2009) Rickettsial outer-membrane protein B (rOmpB) mediates bacterial invasion through Ku70 in an actin, c-Cbl, clathrin and caveolin 2-dependent manner. Cell Microbiol 11: 629–644. 10.1111/j.1462-5822.2008.01279.x 19134120 PMC2773465

[92] Chan YG, Riley SP, Martinez JJ (2010) Adherence to and invasion of host cells by spotted Fever group rickettsia species. Front Microbiol 1: 139 10.3389/fmicb.2010.00139 21687751 PMC3109342

[93] Hillman RD Jr, Baktash YM, Martinez JJ (2013) OmpA-mediated rickettsial adherence to and invasion of human endothelial cells is dependent upon interaction with alpha2beta1 integrin. Cell Microbiol 15: 727–741. 10.1111/cmi.12068 23145974 PMC3610814

[94] Riley SP, Goh KC, Hermanas TM, Cardwell MM, Chan YG, et al (2010) The Rickettsia conorii autotransporter protein Sca1 promotes adherence to nonphagocytic mammalian cells. Infect Immun 78: 1895–1904. 10.1128/IAI.01165-09 20176791 PMC2863548

[95] Kari L, Southern TR, Downey CJ, Watkins HS, Randall LB, et al (2014) Chlamydia trachomatis Polymorphic Membrane Protein D Is a Virulence Factor Involved in Early Host-Cell Interactions. Infect Immun 82: 2756–2762. 10.1128/IAI.01686-14 24733093 PMC4097629

[96] Wuppermann FN, Molleken K, Julien M, Jantos CA, Hegemann JH (2008) Chlamydia pneumoniae GroEL1 protein is cell surface associated and required for infection of HEp-2 cells. J Bacteriol 190: 3757–3767. 10.1128/JB.01638-07 18310329 PMC2394982

[97] Moelleken K, Hegemann JH (2008) The Chlamydia outer membrane protein OmcB is required for adhesion and exhibits biovar-specific differences in glycosaminoglycan binding. Mol Microbiol 67: 403–419. 18086188 10.1111/j.1365-2958.2007.06050.x PMC2229832

[98] Ting LM, Hsia RC, Haidaris CG, Bavoil PM (1995) Interaction of outer envelope proteins of Chlamydia psittaci GPIC with the HeLa cell surface. Infect Immun 63: 3600–3608. 7642297 10.1128/iai.63.9.3600-3608.1995 PMC173500

[99] Su H, Watkins NG, Zhang YX, Caldwell HD (1990) Chlamydia trachomatis-host cell interactions: role of the chlamydial major outer membrane protein as an adhesin. Infect Immun 58: 1017–1025. 2318528 10.1128/iai.58.4.1017-1025.1990 PMC258576

[100] Shimoji Y, Ng V, Matsumura K, Fischetti VA, Rambukkana A (1999) A 21-kDa surface protein of Mycobacterium leprae binds peripheral nerve laminin-2 and mediates Schwann cell invasion. Proc Natl Acad Sci U S A 96: 9857–9862. 10449784 10.1073/pnas.96.17.9857 PMC22300

[101] Govender VS, Ramsugit S, Pillay M (2014) Mycobacterium tuberculosis adhesins: potential biomarkers as anti-tuberculosis therapeutic and diagnostic targets. Microbiology. 10.1099/mic.0.082206-0 25009234

[102] Schorey JS, Li Q, McCourt DW, Bong-Mastek M, Clark-Curtiss JE, et al (1995) A Mycobacterium leprae gene encoding a fibronectin binding protein is used for efficient invasion of epithelial cells and Schwann cells. Infect Immun 63: 2652–2657. 7790081 10.1128/iai.63.7.2652-2657.1995 PMC173355

[103] Ha NY, Cho NH, Kim YS, Choi MS, Kim IS (2011) An autotransporter protein from Orientia tsutsugamushi mediates adherence to nonphagocytic host cells. Infect Immun 79: 1718–1727. 10.1128/IAI.01239-10 21282412 PMC3067549

[104] Lee JH, Cho NH, Kim SY, Bang SY, Chu H, et al (2008) Fibronectin facilitates the invasion of Orientia tsutsugamushi into host cells through interaction with a 56-kDa type-specific antigen. J Infect Dis 198: 250–257. 10.1086/589284 18500929

[105] Li F, Wilkins PP, Crawley S, Weinstein J, Cummings RD, et al (1996) Post-translational modifications of recombinant P-selectin glycoprotein ligand-1 required for binding to P- and E-selectin. The Journal of biological chemistry 271: 3255–3264. 8621728

[106] Miller DP, McDowell JV, Bell JK, Marconi RT (2011) Crystallization of the factor H-binding protein, FhbB, from the periopathogen Treponema denticola. Acta Crystallogr Sect F Struct Biol Cryst Commun 67: 678–681. 10.1107/S1744309111011298 21636910 PMC3107141

[107] O'Boyle NM, Banck M, James CA, Morley C, Vandermeersch T, et al (2011) Open Babel: An open chemical toolbox. J Cheminform 3: 33 10.1186/1758-2946-3-33 21982300 PMC3198950

[108] Carlyon JA, Chan WT, Galan J, Roos D, Fikrig E (2002) Repression of rac2 mRNA expression by Anaplasma phagocytophila is essential to the inhibition of superoxide production and bacterial proliferation. J Immunol 169: 7009–7018. 12471136 10.4049/jimmunol.169.12.7009

[109] Altschul SF, Gish W, Miller W, Myers EW, Lipman DJ (1990) Basic local alignment search tool. J Mol Biol 215: 403–410. 2231712 10.1016/S0022-2836(05)80360-2

[110] Dunning Hotopp JC, Lin M, Madupu R, Crabtree J, Angiuoli SV, et al (2006) Comparative genomics of emerging human ehrlichiosis agents. PLoS Genet 2: e21 16482227 10.1371/journal.pgen.0020021 PMC1366493

[111] Granquist EG, Bardsen K, Bergstrom K, Stuen S (2010) Variant -and individual dependent nature of persistent Anaplasma phagocytophilum infection. Acta Vet Scand 52: 25 10.1186/1751-0147-52-25 20398321 PMC2859769

[112] Thompson JD, Higgins DG, Gibson TJ (1994) CLUSTAL W: improving the sensitivity of progressive multiple sequence alignment through sequence weighting, position-specific gap penalties and weight matrix choice. Nucleic Acids Res 22: 4673–4680. 7984417 10.1093/nar/22.22.4673 PMC308517

