# Supplementary figures and images for: Essential Domains of Anaplasma phagocytophilum Invasins Utilized to Infect Mammalian Host Cells
Source: PLoS Pathog. 2015 Feb 6;11(2):e1004669. doi: 10.1371/journal.ppat.1004669 (PMC4450072; doi:10.1371/journal.ppat.1004669)

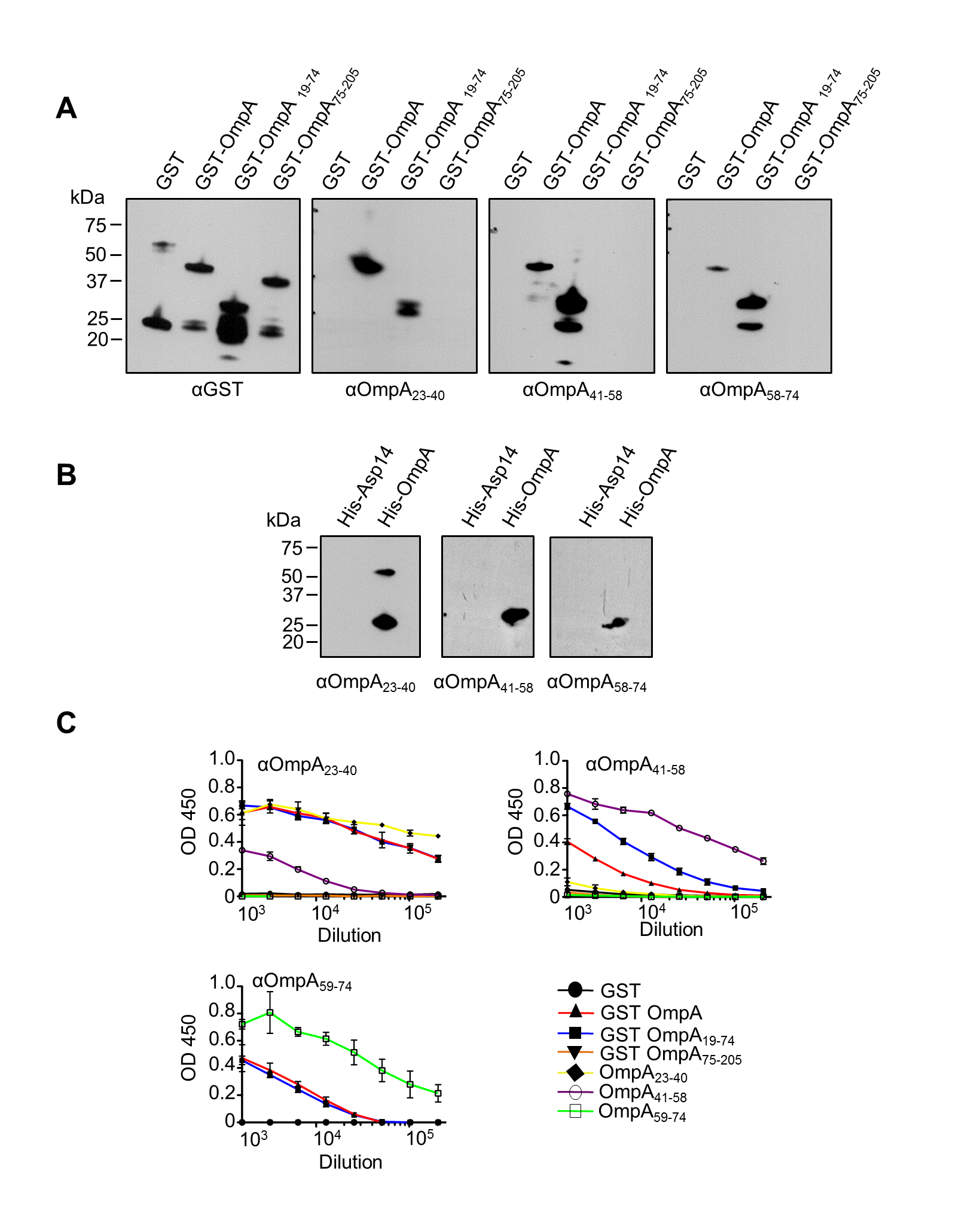

Supplement: S1 Fig — Antibodies raised against peptides corresponding to OmpA23–40, OmpA41–58, and OmpA59–74 were used to screen Western-blotted GST-tagged OmpA, OmpA19–74, OmpA75–205, and GST alone (A) or Western-blotted His-OmpA or His-Asp14 (B) to confirm that each antibody was specific for the recombinant forms of OmpA that contained the target peptide sequences. (C) ELISA in which OmpA23–40, OmpA41–58, and OmpA59–74 antibodies were serially diluted two-fold from 1:200 to 1:409,600 and used to screen wells coated with GST, GST-OmpA, GST-OmpA19–74, GST-OmpA75–205, or peptides corresponding to OmpA23–40, OmpA41–58, or OmpA59–74. Results shown are representative of three independent experiments with similar results. (TIF) [file ppat.1004669.s001.tif]

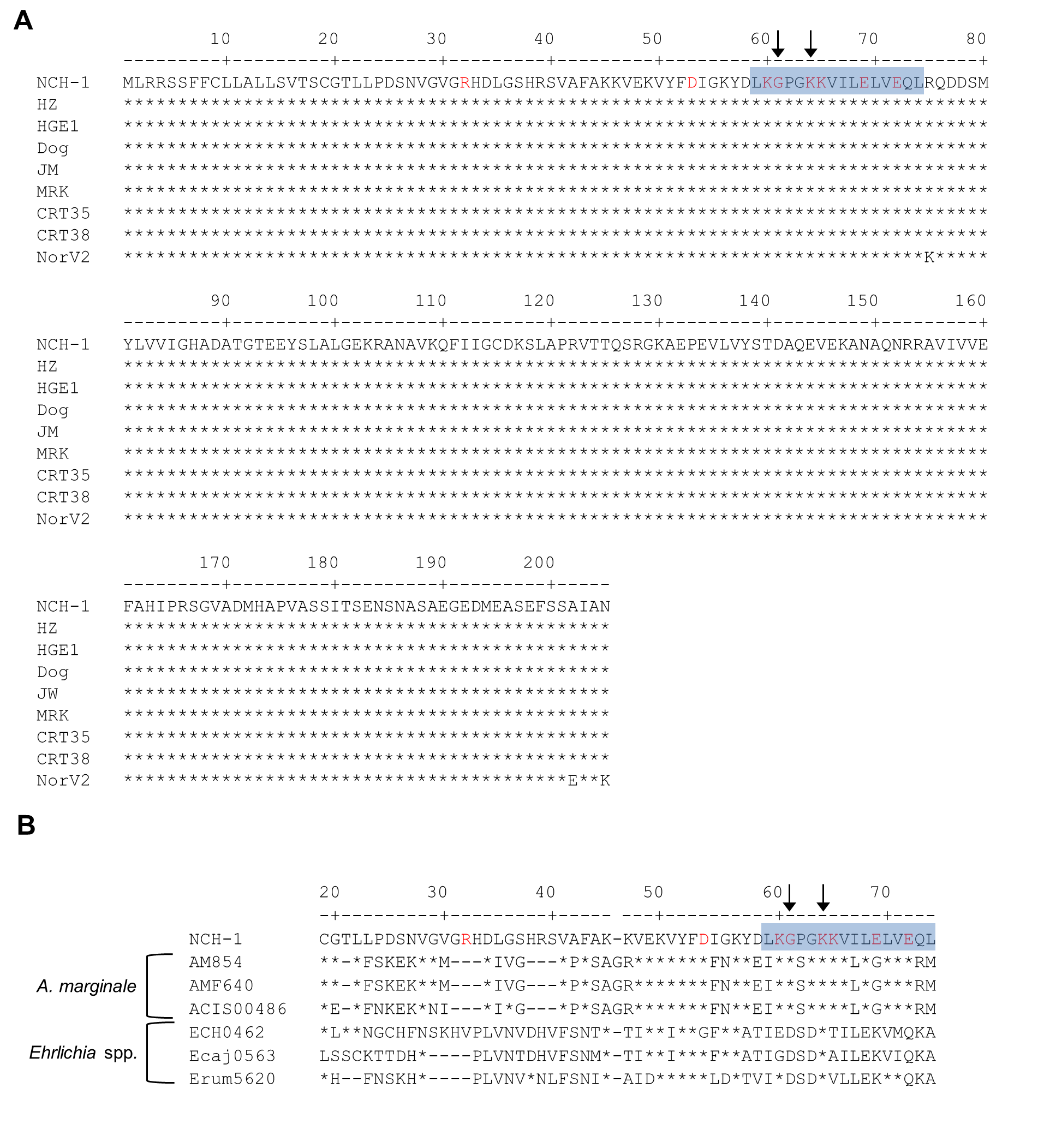

Supplement: S2 Fig — (A) Alignment of OmpA amino acid sequence from the A. phagocytophilum NCH-1 strain (isolated from a human patient in Massachusetts), with OmpA sequences from A. phagocytophilum strains HZ (human; New York), HGE1 (human; Minnesota), Dog (Minnesota), JM (jumping mouse; Minnesota), MRK (horse; California), ApVar-1 isolates CRT35 and CRT38 (both from ticks; Minnesota), and NorV2 (lamb; Norway). (B) Alignment of NCH-1 OmpA amino acids 19 to 74 with corresponding regions of OmpA homologs from the A. marginale St. Maries strain (AM854), A. marginale Florida strain (AMF640), A. marginale subsp. centrale Israel starin (ACIS00486) E. chaffeensis Arkansas strain (ECH0462), Ehrlichia canis Jake strain (Ecaj0563), and the Ehrlichia ruminantium Welgevonden strain (Erum5620). The binding domain corresponding to NCH-1 OmpA residues 59 to 74 is highlighted with blue in (A) and (B). Red text in (A) and (B) denotes amino acids that were mutated to alanine for the experiments presented in Fig. 3 panels B to D. Numbers above the alignments in (A) and (B) denote amino acid position numbers. The arrows in (A) and (B) denote A. phagocytophilum OmpA G61 and K64, which were predicted to form interactions with sLex in Fig. 2 panels D and E and were shown to be critical for OmpA to bind to and mediate infection of mammalian host cells in Figs. 1 and 3. (TIF) [file ppat.1004669.s002.tif]

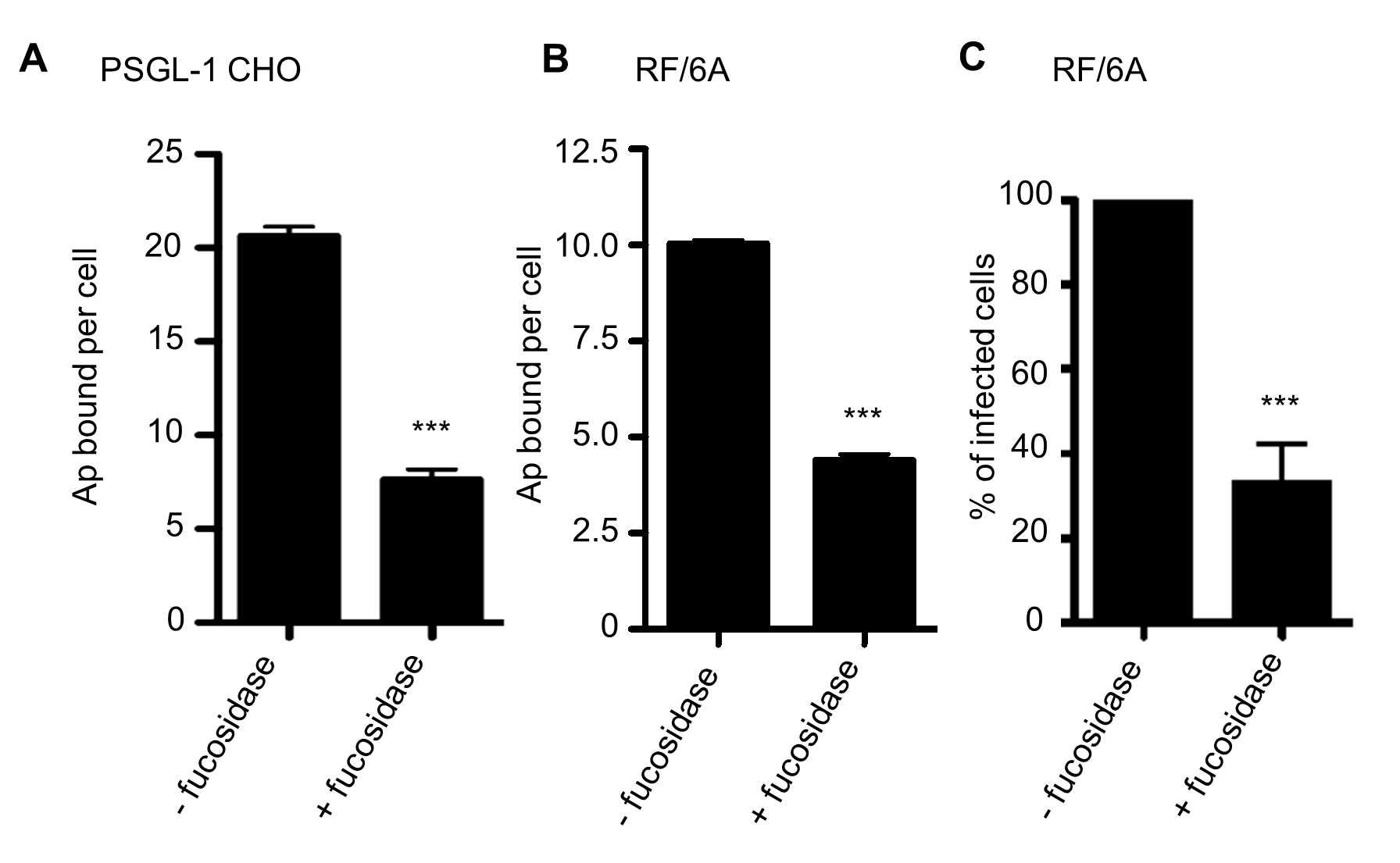

Supplement: S3 Fig — PSGL-1 CHO cells (A) and RF/6A cells (B and C) were treated with α1,3/4-fucosidase (+ fucosidase) or vehicle control (- fucosidase). Fucosidase- and mock-treated cells were incubated with A. phagocytophilum DC organisms. Following the removal of unbound bacteria, the infection of RF/6A cells was allowed to proceed for 24 h prior to being assessed, while bacterial binding to PSGL-1 CHO and RF/6A cells was examined immediately. The mean number (± SD) of bound DC bacteria per PSGL-1 CHO (A) or RF/6A cell (B) or percentage of infected RF/6A cells (C) were determined using immunofluorescence microscopy. Results shown are the means ± SD for three combined experiments. Statistically significant (***P < 0.001) values are indicated. (TIF) [file ppat.1004669.s003.tif]

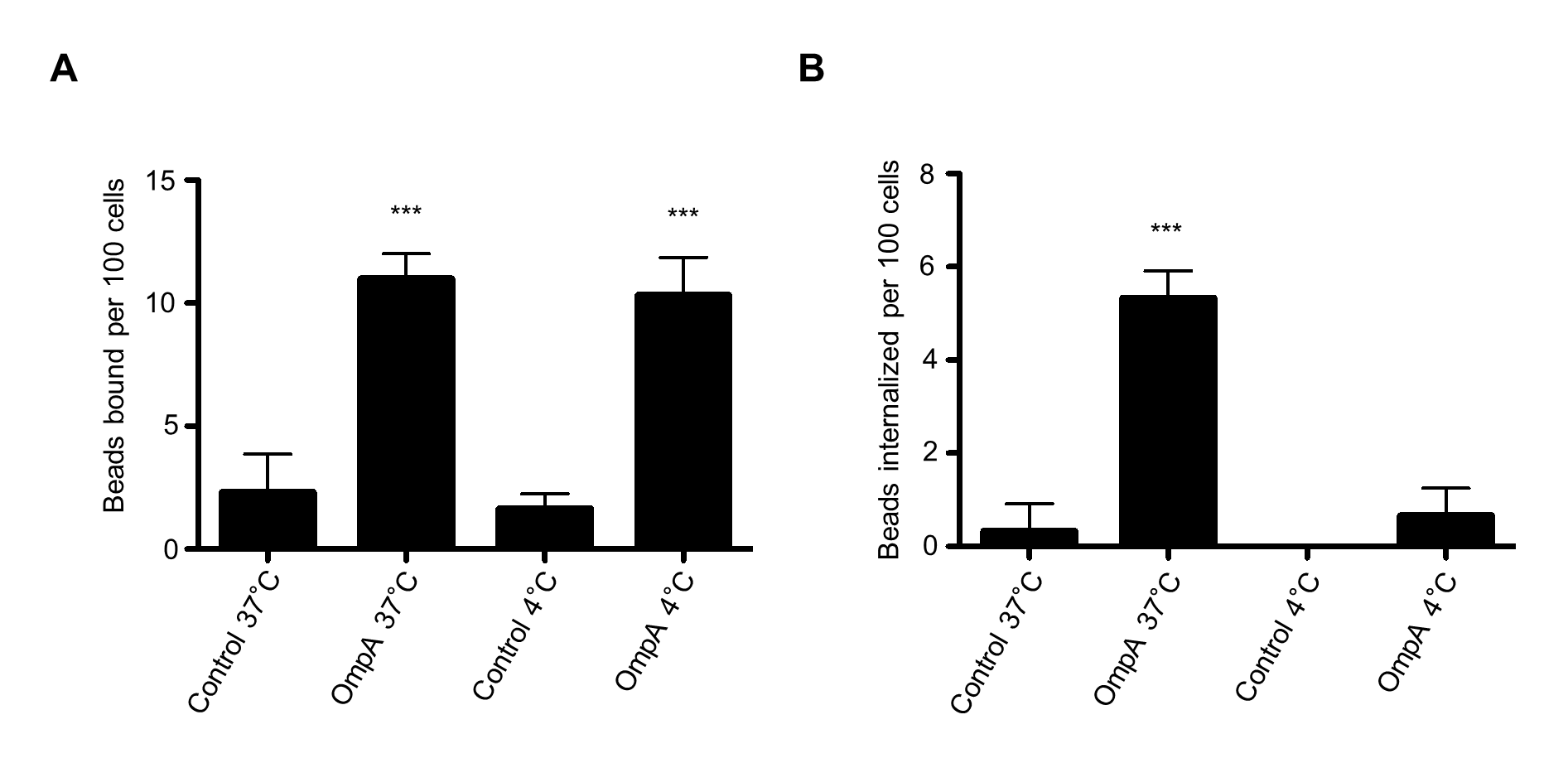

Supplement: S4 Fig — HL-60 cells were incubated with OmpA coated beads or non-coated control beads at 37°C or 4°C. The mean numbers (± SD) of bound (A) and internalized beads (B) were determined using immunofluorescence microscopy. Results presented are representative of three experiments performed in triplicate with similar results. Statistically significant (***P < 0.001) values are indicated. (TIF) [file ppat.1004669.s004.tif]

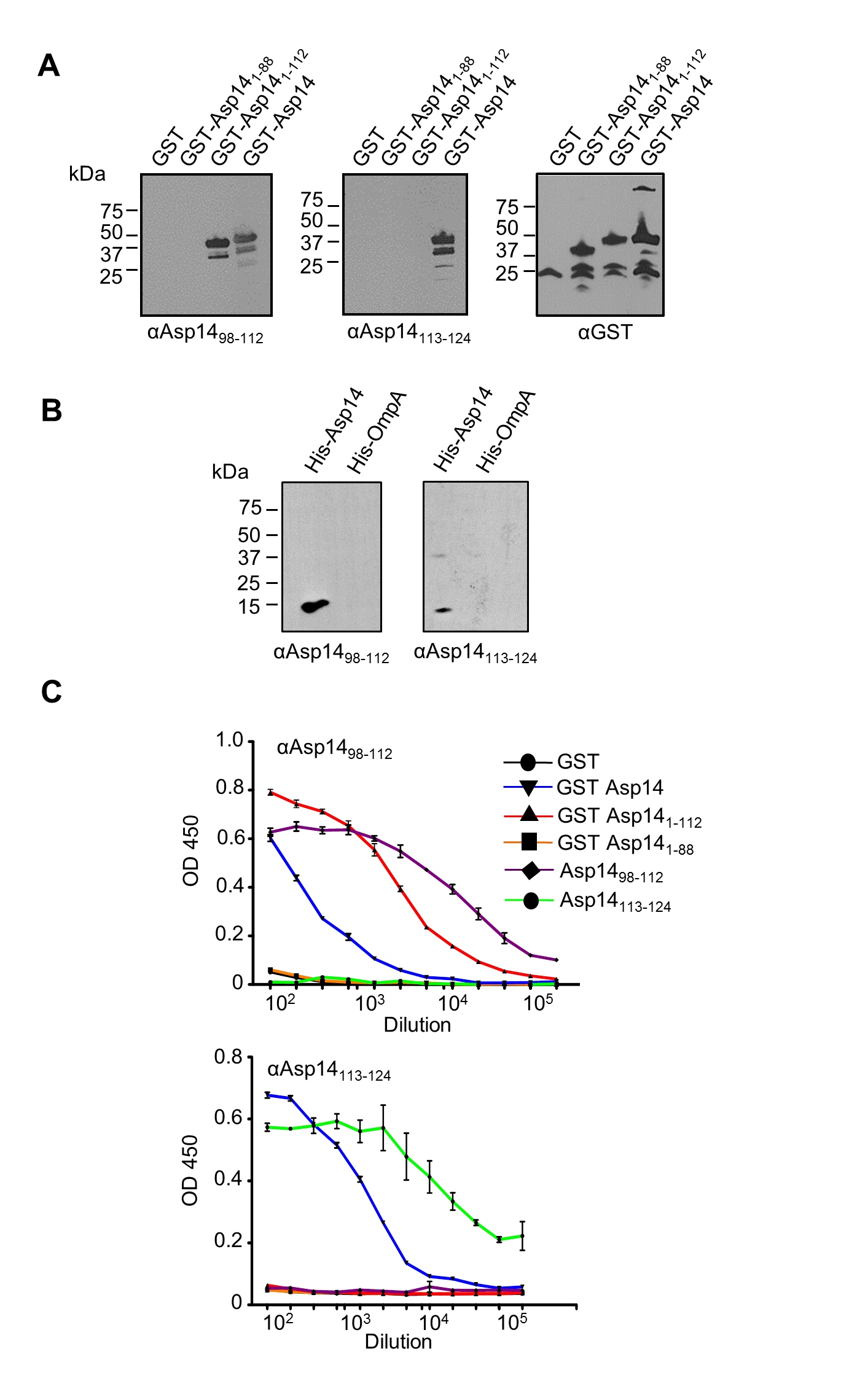

Supplement: S5 Fig — Antibodies raised against peptides corresponding to Asp1498–112 or Asp14113–124 were used to screen Western-blotted GST-Asp14, GST-Asp141–88, GST-Asp141–112, and GST alone (A) or Western-blotted His-Asp14 or His-OmpA (B) to confirm that each antibody was specific for the Asp14 target peptide sequences. (C) ELISA in which serially diluted antibodies raised against Asp1498–112 and Asp14113–124 were used to screen wells coated with GST, GST-Asp14, GST-Asp141–112, GST-Asp141–88, or peptides corresponding to Asp1498–112 or Asp14113–124. Results shown are representative of three independent experiments with similar results. (TIF) [file ppat.1004669.s005.tif]
