# Supplementary material for: Essential Domains of Anaplasma phagocytophilum Invasins Utilized to Infect Mammalian Host Cells
Source: PLoS Pathog. 2015 Feb 6;11(2):e1004669. doi: 10.1371/journal.ppat.1004669 (PMC4450072; doi:10.1371/journal.ppat.1004669)
Supplement: S1 Table — (DOCX) [file ppat.1004669.s006.docx]

| Supplemental Table S1: OmpA oligonucleotides used in this study | | |
| --- | --- | --- |
| Primer Name | Sequence (5' to 3')^a^ | Targeted Nucelotides^b^ |
| Ins_btwn_33-34 | agttggacgtcatgac*tgcctgaaccacctg*cttggaagtcaccgca | 87-118 (+) |
| Ins_btwn_33-34_antisense | tgcggtgacttccaag*caggtggttcaggca*gtcatgacgtccaact | 87-118 (-) |
| Ins_btwn_54-55 | tgagaaagtatatttcgatatc*tgcctgaaccacctg*gggaagtacgatctaaagggtc | 141-184 (+) |
| Ins_btwn_54-55_antisense | gaccctttagatcgtacttccc*caggtggttcaggca*gatatcgaaatatactttctca | 141-184 (-) |
| Ins_btwn_57-58 | cgatatcgggaagtac*tgcctgaaccacctg*gatctaaagggtcccg | 156-187 (+) |
| Ins_btwn_57-58_antisense | cgggaccctttagatc*caggtggttcaggca*gtacttcccgatatcg | 156-187 (-) |
| Ins_btwn_62-63 | gtacgatctaaagggtccc*tgcctgaaccacctg*ggaaagaaggtaattctgg | 168-205 (+) |
| Ins_btwn_62-63_antisense | ccagaattaccttctttcc*caggtggttcaggca*gggaccctttagatcgtac | 168-205 (-) |
| Ins_btwn_67-68 | gggtcccggaaagaaggtaatt*tgcctgaaccacctg*ctggagcttgttg | 180-214 (+) |
| Ins_btwn_67-68_antisense | caacaagctccag*caggtggttcaggca*aattaccttctttccgggaccc | 180-214 (-) |
| Ins_btwn_72-73 | tctggagcttgttgag*tgcctgaaccacctg*cagctcagacaggatg | 201-232 (+) |
| Ins_btwn_72-73_antisense | catcctgtctgagctg*caggtggttcaggca*ctcaacaagctccaga | 201-232 (-) |
| Ins_btwn_77-78 | gagcagctcagacaggat*tgcctgaaccacctg*gacagcatgtatttggt | 214-248 (+) |
| Ins_btwn_77-78_antisense | accaaatacatgctgtc*caggtggttcaggca*atcctgtctgagctgctc | 214-248 (-) |
| R32A | atagtaacgtcggagttggagctcatgaccttggaagtc | 74-112 (+) |
| R32A_antisense | gacttccaaggtcatgagctccaactccgacgttactat | 74-112 (-) |
| D53A | aaaaaagttgagaaagtatatttcgctatcgggaagtacgatctaaag | 133-180 (+) |
| D53A_antisense | ctttagatcgtacttcccgatagcgaaatatactttctcaactttttt | 133-180 (-) |
| G61A | ggaagtacgatctaaaggctcccggaaagaaggta | 164-193 (+) |
| G61A_antisense | taccttctttccgggagcctttagatcgtacttcc | 164-193 (-) |
| GK6164AA | ggaagtacgatctaaaggctcccggagcgaaggtaattctggagct | 164-209 (-) |
| GK6164AA_antisene | agctccagaattaccttcgctccgggagcctttagatcgtacttcc | 164-209 (+) |
| K64A | cgatctaaagggtcccggagcgaaggtaattctggagctt | 171-210 (+) |
| K64A_antisense | aagctccagaattaccttcgctccgggaccctttagatcg | 171-210 (-) |
| K65A | tctaaagggtcccggaaaggcggtaattctggagcttgt | 174-212 (+) |
| K65A_antisense | acaagctccagaattaccgcctttccgggaccctttagat | 174-212 (-) |
| KK6465AA | ggaagtacgatctaaagggtcccggagcggcggtaattctggagcttgttg | 163-214 (+) |
| KK6465AA_antisense | caacaagctccagaattaccgccgctccgggaccctttagatcgtacttcc | 163-214 (-) |
| E69A | gaaagaaggtaattctggcgcttgttgagcagctcag | 188-224 (+) |
| E69A_antisense | ctgagctgctcaacaagcgccagaattaccttctttc | 188-224 (-) |
| E72A | aattctggagcttgttgcgcagctcagacaggatg | 198-232 (+) |
| E72A_antisense | catcctgtctgagctgcgcaacaagctccagaatt | 198-232 (-) |
| ApOmpA_55-LIC | ***gacgacgacaagat***ctgtgggactcttcttccagatagtaacg | 54-82 (+) |
| ApOmpA_618-LIC | ***gaggagaagcccggtta***gttagcgattgcgctagagaattc | 592-618 (-) |
| ^a^Italicized nucleotides denote 15 nucleotide insertions that CLNHL; underlined nucleotides encode alanine substitutions; bold nucleotides denote Ek-LIC cloning sequence.  ^b^(+), positive strand; (-) negative strand. | | |
